# Fiber-shaped aqueous dual-ion batteries integrating rectification and synaptic functions

**DOI:** 10.1093/nsr/nwag062

**Published:** 2026-01-28

**Authors:** Siyuan Ye, Lijie Han, Yu Meng, Long Chen, Yaowu Li, Shan Cong, Guan Wu, Qichong Zhang

**Affiliations:** Key Laboratory of Multifunctional Nanomaterials and Smart Systems, Suzhou Institute of Nano-Tech and Nano-Bionics, Chinese Academy of Sciences, Suzhou 215123, China; National Engineering Lab for Textile Fiber Materials and Processing Technology, Zhejiang Sci-Tech University, Hangzhou 310018, China; Jiangsu Key Laboratory of Organoid Engineering and Precision Medicine, Suzhou Institute of Nano-Tech and Nano-Bionics, Chinese Academy of Sciences, Suzhou 215123, China; Key Laboratory of Multifunctional Nanomaterials and Smart Systems, Suzhou Institute of Nano-Tech and Nano-Bionics, Chinese Academy of Sciences, Suzhou 215123, China; Key Laboratory of Multifunctional Nanomaterials and Smart Systems, Suzhou Institute of Nano-Tech and Nano-Bionics, Chinese Academy of Sciences, Suzhou 215123, China; School of Electrical and Electronic Engineering, Nanyang Technological University, Singapore 639798, Singapore; Key Laboratory of Multifunctional Nanomaterials and Smart Systems, Suzhou Institute of Nano-Tech and Nano-Bionics, Chinese Academy of Sciences, Suzhou 215123, China; Key Laboratory of Multifunctional Nanomaterials and Smart Systems, Suzhou Institute of Nano-Tech and Nano-Bionics, Chinese Academy of Sciences, Suzhou 215123, China; National Engineering Lab for Textile Fiber Materials and Processing Technology, Zhejiang Sci-Tech University, Hangzhou 310018, China; Key Laboratory of Multifunctional Nanomaterials and Smart Systems, Suzhou Institute of Nano-Tech and Nano-Bionics, Chinese Academy of Sciences, Suzhou 215123, China; Jiangsu Key Laboratory of Organoid Engineering and Precision Medicine, Suzhou Institute of Nano-Tech and Nano-Bionics, Chinese Academy of Sciences, Suzhou 215123, China

**Keywords:** fiber-shaped battery, dual-ion battery, energy storage, ionic rectification, neuromorphic synapse, smart textiles

## Abstract

The growing adoption of wearable electronics is spurring the development of lightweight, highly integrable fabric systems. These systems are required to seamlessly merge multiple functions, including energy storage, signal rectification and neuromorphic computing. However, integrating these diverse functionalities into a single fiber structure remains a significant challenge, primarily due to material compatibility issues and distinct operational mechanisms. To address this challenge, we present the novel fiber-shaped aqueous dual-ion batteries (FADIBs) composed of a CuHCF/CNTF cathode, an Ag/CNTF anode and an NH_4_Cl/PVA gel electrolyte. This dual-ion configuration serves as a unified platform that inherently combines these typically disparate functions. Specifically, the FADIBs achieve a high energy density of 51.5 mWh cm^−^^3^ and an exceptional ionic rectification ratio of up to 109, facilitated by asymmetric ion migration. It also emulates artificial synaptic behavior with an ultra-low energy consumption of only 7.5 fJ per synaptic event. Furthermore, the versatility of the FADIBs allows integration into various fabric-based functional modules, demonstrating applications in energy harvesting, power supply and synaptic-controlled electrochromic regulation. This work establishes FADIBs as a foundational technology for multifunctional integration, providing prescient insights for future fabric systems that unify energy management, intelligent perception and information processing.

## Teaser

This innovative fiber merges energy storage, rectification and synaptic functions into one wire, enabling smart fabrics that combine power, logic and adaptive responses for advanced wearable electronics.

## INTRODUCTION

The development of wearable electronics is driving the transition from rigid, discrete components toward flexible, integrated systems [1]. Fabrics have emerged as an ideal platform for this transformation due to their inherently lightweight, flexible and compliant nature, which supports dynamic interaction with the human body [2]. These inherent advantages enable fabrics to transition from passive substrates into active systems capable of integrated energy storage, signal rectification and neuromorphic computing. However, traditional methods often involve assembling discrete functional components into fabrics, resulting in increased bulkiness, decreased flexibility and complex interconnections [3]. Despite advances in discrete batteries [4], ionic diodes [5] and artificial synapses [6], their monolithic integration has been elusive because their functions rely on distinct mechanisms: ion migration; interfacial charge modulation; and synaptic plasticity. A promising pathway to overcome these limitations is to consolidate multiple functionalities within a single fiber-shaped device. This monolithic approach enables seamless integration into fabrics while preserving wearability [1]. However, realizing such integration demands solutions to fundamental challenges in material compatibility and ion-electron coupling [7].

A reliable energy source is a foundational requirement for smart textiles. This has motivated strong interest in fiber-shaped aqueous batteries, which offer a compelling combination of inherent safety, high ionic conductivity and manufacturing compatibility [8,9]. Nevertheless, conventional aqueous battery systems remain fundamentally limited to energy storage, lacking the capacity to support advanced functions such as signal modulation or adaptive learning. In contrast, aqueous dual-ion batteries (ADIBs) operate on the simultaneous migration of both anions and cations. This mechanism unlocks a richer spectrum of electrochemical dynamics, making ADIBs particularly suited for multifunctional applications beyond mere energy storage [10]. Translating this intrinsic potential of ADIBs into practical multifunctional fiber devices, however, is non-trivial. The primary hurdles stem from inherent conflicts in multifunctional integration, including divergent material requirements, incompatible operational conditions and complex interfacial coupling mechanisms. Addressing these challenges therefore necessitates novel design strategies that can support shared ion/electron transport mechanisms across different operational modes, all while maintaining mechanical robustness and scalable manufacturing feasibility [7].

Herein, we present a novel fiber-shaped ADIB (FADIB) that seamlessly integrates energy storage, ionic rectification and artificial synaptic functions into a single, flexible platform. The FADIB features a Cu hexacyanoferrate (CuHCF)/carbon nanotube fiber (CNTF) cathode, an Ag/CNTF anode and a gel electrolyte containing Cl^−^ and NH_4_^+^ ions, engineered to synergistically regulate Faradaic and non-Faradaic processes. During operation, NH_4_^+^ ions reversibly intercalate into the CuHCF cathode, while Cl^−^ ions participate in redox reactions at the Ag anode, enabling stable energy storage with a high capacity of 70.5 mAh cm^−^^3^. The voltage-dependent ion migration further creates directional charge transport, yielding rectification ratios up to 109. Simultaneously, the gradual accumulation and relaxation of ions under pulsed stimuli emulate synaptic plasticity, allowing the FADIB to replicate key neuromorphic functions such as excitatory/inhibitory postsynaptic currents (EPSCs/IPSCs) and spike-rate-dependent plasticity (SRDP). Beyond its electrochemical versatility, the FADIB’s fiber format ensures mechanical robustness (83.3% capacity retention after 3000 bending cycles) and modular scalability. The FADIB enables direct integration into fabrics for practical applications, such as powering electroluminescent displays and driving electrochromic fabrics with synaptic-controlled color transitions. Furthermore, its rectification properties are harnessed in logic gates and triboelectric nanogenerator systems, highlighting its potential for wearable computation and energy harvesting. This work represents a significant leap toward multifunctional fiber-shaped devices that unify energy, computation and adaptive responses in a single wire. By addressing the integration challenges through dual-ion design and interfacial engineering, the FADIB platform not only overcomes the trade-off between functionality and miniaturization but also opens new avenues for smart fabrics.

## RESULTS AND DISCUSSION

### Structure and working mechanism of FADIBs

**Figure 1.**
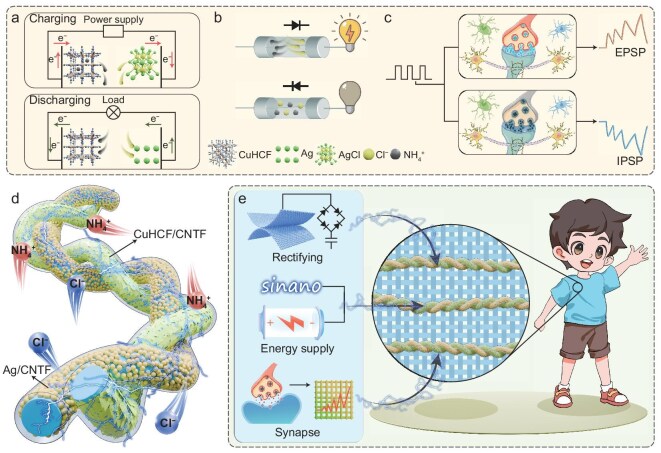
Schematic illustration of the functional mechanisms, structure and applications of FADIBs. (a) Energy storage mechanism. (b) Diode mechanism. (c) Synapse mechanism. (d) Schematic structure. (e) Integration into wearable fabrics enabling flexible energy harvesting, power supply and signal modulation.

Figure 1a–c illustrates the core mechanisms of our designed unique ADIB, which integrates the capabilities of an energy storage battery, an ionic diode and an artificial synapse. Despite these distinct functions, they share a common foundation: operation governed by the modulation of ion migration and interfacial charge. In a typical ADIB, energy storage is achieved through the reversible ion migration between the electrodes during electrochemical redox reactions, with the associated electron transfer driving current through the external circuit [11] (Fig. 1a). Specifically, during the charging process, NH_4_^+^ ions are extracted from the CuHCF cathode, while the AgCl as the anode is electrochemically reduced to Ag. Upon discharging, NH_4_^+^ ions intercalate into the CuHCF framework, accompanied by the oxidation of Ag to form AgCl at the anode. In contrast to traditional rocking-chair batteries, this unique dual-ion mechanism not only enhances the energy storage kinetics but also demonstrates its potential to modulate additional signals, transforming the device from a pure energy source into a multifunctional platform.

Ionic diodes operate based on asymmetric ion transport routes and interfacial processes, which enable unidirectional current flow [12]. Figure 1b elucidates the current rectification mechanism governed by bias-dependent charge-transfer processes. Under forward bias (0 to 1 V), the current stems from both Faradaic reactions, which include the reversible (de)intercalation of NH_4_^+^ at the CuHCF cathode and the Ag/AgCl conversion at the anode, and a significant electric double-layer (EDL) contribution. In contrast, under reverse bias (−1 to 0 V), Faradaic reactions are entirely suppressed, and the EDL current is substantially weaker. This collective asymmetry, enhanced by the dual-ion effects, results in a markedly high rectification ratio, confirming the intrinsic ionic diode behavior of the ADIB. Artificial synapses emulate synaptic plasticity through the time-dependent accumulation and relaxation of ions at the interface (Fig. 1c). Specifically, under electrical stimulation, ion migration and redistribution dynamically modulate the interfacial capacitance and charge carrier density, thereby regulating the postsynaptic current (PSC) in a temporally dependent manner and eliciting excitatory and inhibitory postsynaptic potentials (EPSPs/IPSPs) [13]. The dual-ion configuration further augments this process, where the synergistic roles of cations and anions at their respective interfaces lead to more pronounced ionic relaxation and charge redistribution. This enhancement enables precise temporal signal differentiation and low-voltage operation, paving the way for energy-efficient neuromorphic systems [14]. This ion-mediated dynamic modulation mechanism bears a fundamental resemblance to the efficacy regulation in biological synapses, which relies on the accumulation and clearance of neurotransmitters.

As illustrated in Fig. 1d, the novel FADIB consists of a CuHCF cathode grown on CNTF and an Ag anode deposited on CNTF, separated by an NH_4_Cl/polyvinyl acetate (PVA) gel electrolyte that provides a medium for dual-ion transport. By harnessing the dual-ion mechanism, the FADIB is not only capable of energy storage, but also exhibits ionic rectification and artificial synaptic behavior. These functions are conditionally activated through various voltage regimes, expanding versatility and applicability. As shown in Fig. 1e, FADIBs can be seamlessly integrated into fabric architectures, serving as multifunctional modules in electronic fabric system. Furthermore, they can be easily integrated with other fiber/fabric-based components to enable more complex and versatile functionality. Specifically, the innovative ionic rectification behavior is combined with triboelectric nanogenerators (TENGs) to form self-powered energy harvesting modules that consistently ensure a single direction of current flow and efficient energy transfer. The remarkable energy storage capacity of FADIBs makes them perfect for serving as on-body power sources, efficiently driving electroluminescent displays and delivering a reliable, flexible energy supply. In addition, the synaptic plasticity-inspired ionic modulation enables dynamic control of electrochromic fabrics, allowing color transitions in the fabrics to be controlled by their previous stimulation history, becoming a pivotal aspect for developing adaptive and interactive visual interfaces. These demonstrations highlight the immense potential of FADIBs to become integrated multifunctional nodes in next-generation wearable platforms, enabling the convergence of energy storage, signal processing and adaptive response within fabric systems.

### Material characterization and structural analysis

**Figure 2.**
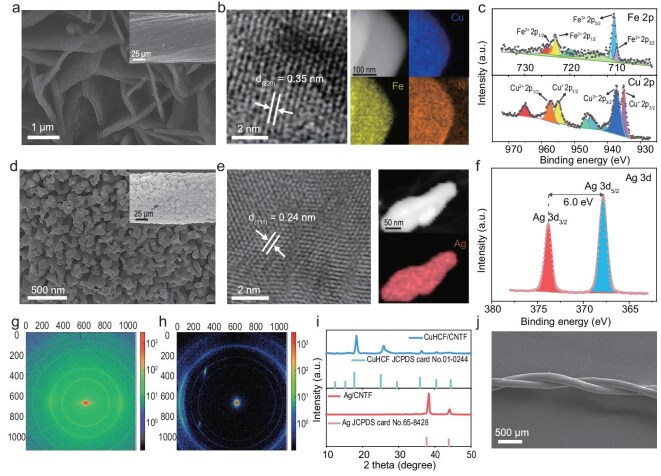
Fabrication and characterization of CuHCF/CNTF and Ag/CNTF. (a) SEM images of CuHCF/CNTF. (b) HRTEM and EDX images of CuHCF/CNTF. (c) Fe 2p and Cu 2p XPS spectra of CuHCF/CNTF. (d) SEM images of Ag/CNTF. (e) HRTEM image and EDX elemental mapping of Ag/CNTF. (f) Ag 3d XPS spectrum of Ag/CNTF. 2D WAXD pattern collected from (g) CuHCF/CNTF and (h) Ag/CNTF. (i) 1D WAXD curves of CuHCF/CNTF and Ag/CNTF. (j) SEM image of the FADIBs.

CuHCF/CNTF was crafted through a step-by-step synthesis process on the CNTF substrate. Scanning electron microscopy (SEM) images at different synthesis stages reveal the morphological evolution. Initially, dense and uniform Cu nanoparticles were electrodeposited onto the CNTF surface (Fig. S1a–c). Subsequent oxidation shifted these surface Cu nanoparticles into vertically aligned CuO nanosheets (Fig. S1d–f), forming a structural template. Ultimately, via a combined etching process, neat and distinct CuHCF nanosheets were achieved (Fig. 2a). High-resolution transmission electron microscopy (HRTEM) images further illustrate well-defined lattice fringes with an interplanar spacing of approximately 0.35 nm, which aligns with the (220) plane of CuHCF (Fig. 2b). This precision is further underscored by energy-dispersive X-ray (EDX) spectroscopy findings, which indicate a uniform distribution of Fe, Cu, and N elements within CuHCF, confirming the homogeneous deposition of its components. X-ray photoelectron spectroscopy (XPS) spectra (Fig. 2c and Fig. S2) reveal the coexistence of Cu^+^/Cu^2+^ and Fe^2+^/Fe^3+^ species, reflecting the mixed-valency nature of the CuHCF framework. This mixed valency is further supported by Raman spectra (Fig. S3), which exhibit a prominent C≡N stretching vibration peak at approximately 2090 cm^−^^1^, confirming the presence of the Fe–C≡N–Cu coordination framework [15]. Additionally, the D band (~1345 cm^−^^1^), G band (~1580 cm^−^^1^) and G′ band (~2700 cm^−^^1^) are distinct features of CNTF, further highlighting its integration within the final structure. The synthesis of Ag nanoparticles through electrochemical deposition, as illustrated in Fig. 2d, results in a uniformly distributed particulate structure on the surface of Ag/CNTF, indicating the successful deposition of the Ag component onto the CNTF substrate. HRTEM images in Fig. 2e exhibit well-defined lattice fringes with a spacing of 0.24 nm, which corresponds to the (111) plane of metallic Ag. EDX mapping demonstrates a uniform distribution of Ag, suggesting a stable and homogeneous structure. Furthermore, XPS analysis in Fig. 2f reveals characteristic Ag 3d_5/2_ and Ag 3d_3/2_ peaks at 368.1 and 374.1 eV, consistent with metallic Ag binding energies, confirming the successful synthesis of Ag/CNTF [16].

To clarify the surface phase state of CuHCF/CNTF and Ag/CNTF, wide-angle X-ray scattering (WAXS) measurements were conducted to further elucidate the structural evolution during the synthesis process. As shown in the 2D WAXS patterns (Fig. 2g and h), CuHCF/CNTF exhibits uniform and well-defined concentric diffraction rings, indicative of high crystallinity and homogeneous grain distribution, whereas Ag/CNTF displays arc-shaped diffraction spots, suggesting partial preferred orientation and particle size variation while still well maintaining crystallinity. These observations are further corroborated by the corresponding 1D WAXS profiles (Fig. 2i), where sharp diffraction peaks at approximately 17.4°, 24.8° and 35.4° are assigned to the (200), (220) and (400) planes of cubic-phase CuHCF/CNTF, confirming the crystalline structure and successful transformation from CuO. The corresponding 1D and 2D WAXS patterns of Cu/CNTF and CuO/CNTF are shown in Fig. S4, further illustrating the structural evolution during the conversion processes. The Ag/CNTF sample shows distinct peaks at 38.4°and 44.1°, corresponding to the (111) and (200) planes of face-centered cubic metallic Ag, thereby validating the formation of crystalline Ag with a well-defined metallic phase (Fig. 2h and i). Tensile stress–strain curves of the CuHCF/CNTF, Ag/CNTF and pristine CNTF fibers were tested to evaluate their mechanical properties (Fig. S5). The results confirm that the fibers maintain good mechanical strength, even after the growth of active materials. Finally, as illustrated by the SEM image in Fig. 2j, the as-prepared CuHCF/CNTF and Ag/CNTF electrodes were uniformly coated with a gel electrolyte and subsequently twisted to form a FADIB.

### Electrochemical performance for energy storage

**Figure 3.**
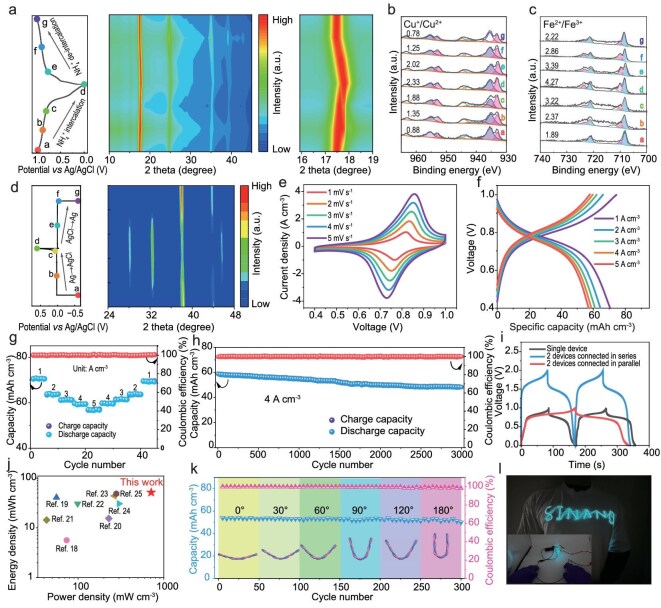
Electrochemical performance of the FADIBs. (a) The GCD curves of the CuHCF/CNTF and corresponding *ex situ* WAXS patterns of CuHCF/CNTF at different charge–discharge states. (b) Fe 2p and (c) Cu 2p XPS spectra of CuHCF/CNTF at various charge–discharge states. (d) The GCD curves of the Ag/CNTF and corresponding *ex situ* WAXS patterns of Ag/CNTF at diverse charge–discharge states. (e) CV curves, (f) GCD curves and (g) rate performance of the FADIBs. (h) Cycling performance of the FADIBs at 4 A cm^−^^3^. (i) GCD curves of a single FADIB and two FADIBs connected in series and parallel. (j) Ragone plot comparing energy and power densities. (k) Capacitance retention under bending at different angles. (l) Demonstration of our FADIBs lighting up LED arrays.

The electrochemical performance of the CuHCF/CNTF and Ag/CNTF electrode were evaluated using a three-electrode system. As shown in Fig. S6, the cyclic voltammetry (CV) curves of CuHCF/CNTF exhibit distinct redox peaks, with the main oxidation and reduction peaks located at approximately 0.82 and 0.81 V, indicating highly reversible intercalation/deintercalation behavior of NH_4_^+^. Galvanostatic charge–discharge (GCD) measurements show stable voltage plateaus under various current densities, delivering a capacity of 119.7 mAh cm^−3^ at a current density of 1 A cm^−3^. To investigate the structural evolution of the CuHCF/CNTF cathode during charge–discharge processes, a series of characterization techniques were employed to analyze its chemical composition and elucidate the underlying energy storage mechanism. Seven representative states during the charge–discharge cycle were selected for *ex situ* measurements. As shown in Fig. 3a, the *ex situ* WAXS results reveal that the main diffraction peak corresponding to the (200) plane of CuHCF shifts to higher angles during discharging and returns to its original position upon charging, indicating a reversible insertion/extraction of NH_4_^+^. Notably, no new diffraction peaks were observed throughout cycling, suggesting that the crystalline structure of CuHCF remains stable. In addition, *ex situ* XPS spectra further elucidate alterations in the valence states of Fe 2p and Cu 2p at specific voltage states, providing insights into the NH_4_^+^ storage mechanism in CuHCF (Fig. 3b and c). It is evident that during the transition from state a to state d, with the insertion of NH_4_^+^ during the discharge process, the Fe sites and Cu sites both participate in the reaction, resulting in an increase of Fe(II)/Fe(III) and Cu(I)/Cu(II) ratios. Conversely, from state d to state g, the Fe (II)/Fe (III) Cu(I)/Cu(II) ratios gradually return to their initial state as NH_4_^+^ ions are extracted during the charging stage. The results clearly confirm that both Cu and Fe serve as active redox centers, cooperatively participating in Faradaic reactions and enabling dual electron transfer pathways [17]. This synergistic effect significantly enhances the electrochemical performance and energy storage efficiency of the electrode material.

As shown in Fig. S7a, the Ag/CNTF electrode displays clear redox peaks in the CV curves at various scan rates, corresponding to the reversible conversion between Ag and AgCl. The GCD curves (Fig. S7b) exhibit stable voltage plateaus across different current densities, confirming good electrochemical reversibility, with a capacity of 1292.9 mAh cm^−3^ delivered at 1 A cm^−3^. To further investigate the structural evolution of the Ag/CNTF electrode during the charge–discharge process, *ex situ* WAXS characterization was performed at seven selected states, as shown in Fig. 3d. During the charging process (from −0.60 to 0.60 V), the intensity of the diffraction peak at 38.1°, corresponding to the (111) plane of metallic Ag, gradually decreases, indicating the oxidation of Ag to AgCl. In the subsequent discharging process (from 0.60 back to −0.60 V), the peak intensity recovers, suggesting the reduction of AgCl back to Ag. No new diffraction peaks were observed, and the peak position remained unchanged throughout, signifying a highly reversible Ag/AgCl conversion process.

FADIBs were assembled using a CuHCF/CNTF cathode, an Ag/CNTF anode and an NH_4_Cl/PVA gel electrolyte, with their energy storage mechanism illustrated in Fig. S8. As shown in Fig. 3e, the CV curves of the FADIBs recorded at various scan rates (1–5 mV s^−1^) exhibit distinct and symmetric redox peaks. The GCD profiles depicted in Fig. 3f, obtained at various current densities ranging from 1 to 5 A cm^−^^3^, exhibit clear voltage plateaus, indicating the consistent charge–discharge behavior of the FADIBs. At 1 A cm^−^^3^, the FADIBs deliver a capacity of 70.5 mAh cm^−3^. The Nyquist plot (Fig. S9) reveals a small semicircle in the high-frequency region, indicating low internal resistance. As illustrated in Fig. 3g, when the current density increases from 1 to 5 A cm^−3^, the FADIBs maintain a capacity of 56.8 mAh cm^−^^3^, corresponding to a capacity retention of 80.6%. Notably, when the current density returns to 1 A cm^−3^, the capacity recovers to 98.2% of its initial value, highlighting the outstanding rate capability and reversibility of the FADIBs.

As shown in Fig. 3h, the FADIBs demonstrate excellent cycling stability over 3000 charge–discharge cycles at a current density of 4 A cm^−3^, retaining 82.1% of their initial capacity with nearly 100% Coulombic efficiency, indicating outstanding long-term durability and structural integrity. In order to enhance voltage and capacity outputs, Fig. 3i illustrates the strategic integration of individual FADIBs in both series and parallel configurations. The results clearly indicate that these configurations can effectively boost the output voltage and current while maintaining minimal performance loss. Furthermore, our calculations reveal a significant energy density of 51.5 mWh cm^−3^, accompanied by a power density of 713.3 mW cm^−3^. This performance, evident from Fig. 3j and Table S1, surpasses most previously reported flexible fiber-shaped batteries [18–25], highlighting the exceptional energy storage capability of FADIBs. As depicted in Fig. 3k, FADIBs maintain stable capacity and high capacity retention when bent from 30° to 180°, demonstrating exceptional mechanical flexibility for practical energy storage. Finally, the FADIBs successfully powered an electroluminescent thread, which can be woven into textiles to illuminate a predefined pattern, demonstrating their strong potential for integration into wearable electronics (Fig. 3l).

### Ionic rectification property and applications

**Figure 4.**
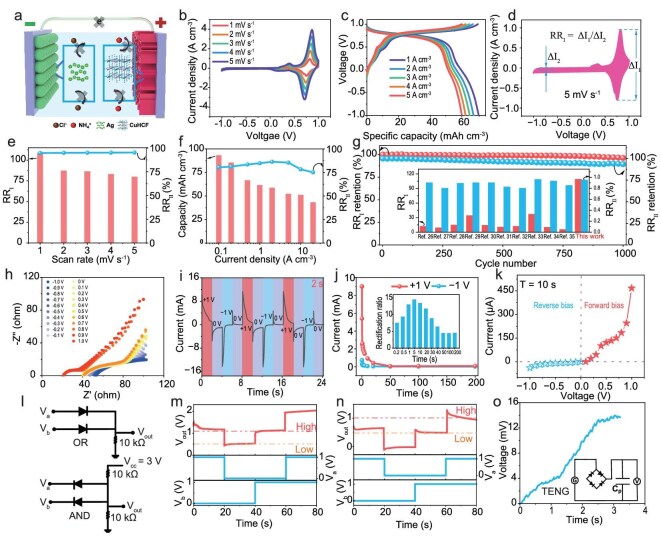
Rectification performance of the FADIBs. (a) Schematic of ion/electron transport under reverse bias. (b) CV curves at various scan rates. (c) GCD curves at different current densities. (d) Schematic illustrating the RR_I_ calculation method. (e) RR_I_ and RR_II_ values at different scan rates. (f) Specific capacity and RR_I_ at different current densities. (g) Cyclic RR_I_ and RR_II_ at 5 mV s^−^^1^ (inset: performance comparison with other rectifying devices). (h) Nyquist plots under applied DC bias. (i) I–t curve for an external bias alternating between +1 and −1 V. (j) Steady-state current under +1 V forward and −1 V reverse bias at specified durations (inset: corresponding rectification ratio). (k) Current response at different applied biases (10 s pulse). (l) Circuit diagrams for ionic logic gates. Operational characteristics of ionic (m) OR and (n) AND gates, respectively. (o) Output voltage measurements (inset: the corresponding circuit diagram).

As illustrated in Fig. 4a, the rectification device was constructed based on the operating principle of FADIBs. Specifically, during the forward bias (0–1 V), the Faradaic reactions are caused by the deintercalation of NH_4_^+^ ions from the CuHCF cathode and the redox conversion of Ag at the anode. In contrast, under reverse bias (−1 to 0 V), no Faradaic reactions occur, and current flow is largely suppressed. Two rectification ratios were calculated to evaluate the rectification performance of FADIBs. Rectification ratio I (RR_I_) is defined as the ratio of the maximum current in the positive voltage range to the minimum current in the negative voltage range during CV testing (Fig. 4b–d). Rectification ratio II (RR_II_) represents the percentage of the capacity in the positive voltage region relative to the total capacity, and can be calculated via both CV and GCD methods (Fig. 4c and Fig. S10). Figure 4e and f depict the variations of both rectification ratios with CV scan rate and GCD current density. Notably, RR_I_ attains a maximum value of 109 at a scan rate of 1 mV s^−^^1^, while RR_II_ remains nearly constant at an average of 94.5% across the 1–5 mV s^−^^1^ range. These results demonstrate the excellent unidirectional conduction of FADIBs. To evaluate the stability during prolonged operation, continuous CV cycling tests were conducted. As shown in Fig. 4g and Fig. S11, the results after 1000 cycles indicate that RR_I_ remained at 93.4% and RR_II_ reached 96.3%, which ensures reliable operation in unidirectional conduction and energy storage. Moreover, as depicted in Fig. 4g and Table S2, a comparison with previously reported supercapacitor-based ionic diode (CAPode) devices highlights the superior rectification performance of FADIBs [26–35], further confirming their potential for dual-ion regulation and rectified applications.

The high rectification ratio of the FADIBs originates from the efficient coupling of Faradaic and non‑Faradaic processes. While the influence of the dual‑ion strategy on the Faradaic reaction has been discussed above, its role in non‑Faradaic migration is elucidated here through systematic zeta potential measurements of CuHCF/CNTF and Ag/CNTF in different electrolytes, including 0.01 M NH_4_Cl, (NH_4_)_2_SO_4_, and KCl (Fig. S12). The results show that the CuHCF//Ag pair demonstrated a notably larger zeta potential difference in NH_4_Cl, suggesting a more pronounced asymmetry in the surface charge distribution. This specific interaction leads to the formation of a built‑in ion gradient, driven by electrode‑ion selective adsorption in the initial state, rather than the uniform distribution typical of conventional systems. The pre‑established interfacial electrostatic gradient can effectively direct the migration of ions along a preferred direction under an applied bias (enabling fast transport under forward bias), thereby synergizing with the Faradaic process to collectively achieve the notably high rectification performance [36].

To further investigate the rectification behavior of FADIBs, electrochemical impedance spectroscopy (EIS) measurements were conducted under various bias voltages. As depicted in Fig. 4h, under forward biases, the Nyquist plots exhibit smaller semicircles yet lower real impedance. This suggests a reduced interfacial charge-transfer resistance and an enhanced charge transport mechanism. Conversely, under reverse biases, the plots reveal larger semicircles and higher impedance, indicating a hindrance in charge transfer. Notably, a significant reduction in solution resistance is also observed when the voltage reaches 0.8 and 1.0 V. These observations collectively demonstrate that the dual-ion strategy exerts a comprehensive influence under bias, modulating not only the interfacial charge-transfer and diffusion processes but also the bulk electrolyte properties. The Bode plots further highlight the frequency response dependent on the bias (Fig. S13). Under forward bias, FADIBs exhibit lower phase angles and higher conductivity in the low-frequency region, suggesting improved charge transport and reduced interfacial capacitance. Conversely, reverse bias yields higher phase angles and greater impedance, indicating increased interfacial polarization and restricted charge injection [5]. This pronounced low-frequency asymmetry strongly validates the electrochemical basis of the high macroscopic rectification ratio.

In order to explore the potential of the fiber-shaped diode for practical logic operations and data processing, a series of current response tests was conducted. As shown in Fig. S14, the current–time (I–t) curves under alternating +1 and −1 V bias at various modulation durations reveal dynamic response of FADIBs to periodic voltage inputs. Figure4i and Fig. S15 introduce a 0 V interval between the alternating bias voltages, which effectively minimizes the polarization interference caused by the continuous application of the bias. Under alternating bias, FADIBs exhibit distinct rectifying behavior, with forward current markedly exceeding reverse current, confirming their asymmetric ion transport properties. Subsequently, the final current decay values at different bias durations were analyzed (Fig. S16 and Fig. 4j), and the corresponding rectification ratios under +1 and −1 V were calculated, providing a quantitative assessment of the FADIBs’ rectification performance. The rectification ratios exhibit a trend of initially increasing and then decreasing, reaching a peak value of 14 at 5 s. Especially, when the duration extends beyond 50 s, the rectification ratio stabilizes at approximately 4 without significant fluctuation. This suggests that FADIBs exhibit a consistent rectifying behavior even during prolonged operation, which is crucial for their practical applications in sustained logic computation and wearable electronics. Figure S17 and Fig. 4k present the current responses of the FADIBs under different applied voltages ranging from −1 to +1 V, with each voltage maintained for 10 s. As the applied voltage increases, the forward current rises significantly, while the reverse current remains consistently low. Notably, the voltages at which the current increases sharply (0.3 and 0.9 V) correspond to the onset of oxidation reactions in FADIBs, further confirming that the high current observed in the forward bias region is closely associated with Faradaic processes.

To evaluate the feasibility of FADIBs for logic circuit applications, we constructed basic OR and AND gates utilizing their asymmetric ion transport behavior. The corresponding circuit diagram is shown in Fig. 4l. Four input states, (0, 0), (0, 1), (1, 0) and (1, 1), were applied to nodes a and b, and the output signal at node OUT was recorded. The output voltage (V_out_) of the OR gate is shown in Fig. 4m. When either input a or b was in the ‘1’ state (1 V), V_out_ remained at a high level, corresponding to the red dashed line (~1.1 V). Conversely, when both inputs were ‘0’, V_out_ dropped to a low level (~0.5 V), indicated by the orange dashed line, representing a logic ‘0’. Figure4n displays the output of the AND gate. When either input was ‘0’, the corresponding diode turned on due to an external bias (V_cc_), leading to current flow and a low output (~0.6 V), shown by the orange dashed line. When both a and b were ‘1’, the diodes remained off due to insufficient bias to trigger conduction, yielding a high output (~0.95 V), indicated by the red dashed line. These results confirm the FADIBs’ stable logic response and effective rectification, highlighting their potential for advanced ionic logic circuits and integrated systems. In order to evaluate the rectification capability of the device in practical AC energy harvesting systems, we incorporated them into a bridge rectifier circuit and connected them to a fabric-based TENG, as depicted in Fig. 4o. The inset circuit diagram illustrates a full-wave bridge rectifier, comprising four rectifying FADIBs, along with an external parallel capacitor for charge accumulation. The output voltage gradually escalated to approximately 13 mV within a span of 3 s, demonstrating efficient rectification and charging by the FADIBs, which confirms their ability to convert TENG AC output into a reliable DC voltage for wearable energy storage.

### Synaptic plasticity emulation and neuromorphic applications

**Figure 5.**
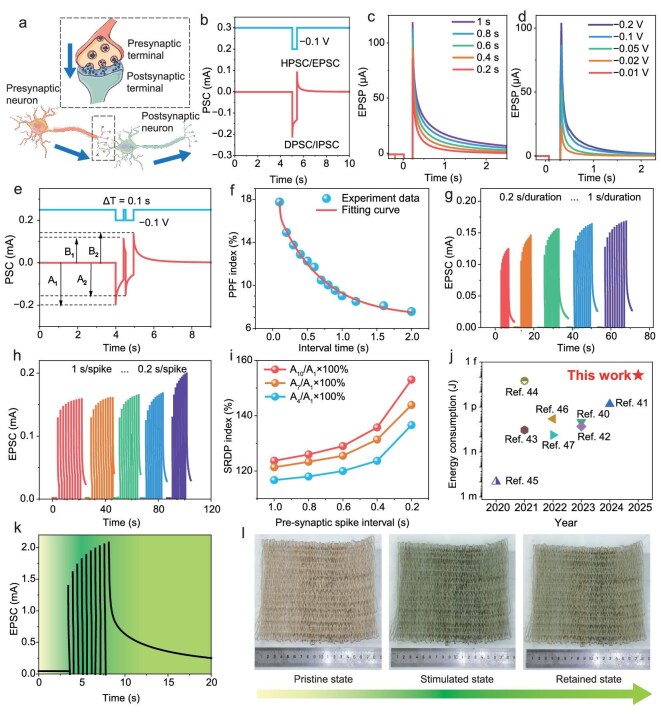
Artificial synaptic characteristics of the FADIBs. (a) Schematic of biological synaptic transmission via neurotransmitter release. (b) EPSC, IPSC, DPSC and HPSC triggered by a negative presynaptic spike. Spike-duration-dependent plasticity modulated by varying (c) spike width and (d) amplitude. (e) Pulse facilitation behaviors. (f) PPF index. EPSC response to 10 consecutive spikes at (g) different durations and (h) frequencies. (i) SRDP index versus stimulation frequency. (j) Energy consumption comparison with previously reported artificial synapses. (k) EPSC response to consecutive spikes at V = −1 V. (l) Electrochromic fabric modulation demonstration.

The inherent ionic gradient engineered by the dual-ion strategy serves as the foundation for the voltage-sensitive migration and interfacial dynamics in FADIBs. Extending beyond energy storage and rectification, these dynamics open avenues for mimicking synaptic behaviors in neuromorphic systems. In such ion-based devices, synapse-like information processing is achieved through electrolyte relaxation behavior [37]. This emulation parallels biological synapses, where signal transmission relies on precise neurotransmitter release to induce postsynaptic depolarization (Fig. 5a) [38]. To evaluate the synaptic behavior of FADIBs, we conducted simulations of ion migration processes and documented the corresponding PSC responses. The results, depicted in Fig. 5b, clearly show that when an external electric field is applied with a negative presynaptic pulse (−0.1 V for 0.4 s), FADIBs exhibit a significant increase in PSC, resembling a depolarized PSC (DPSC). After the pulse is withdrawn, ions gradually redistribute under the influence of the internal electric field, resulting in a negative PSC that mimics a hyperpolarized PSC (HPSC). This bidirectional PSC response is also observed under positive voltage stimuli (+0.1 V, 0.4 s) (Fig. S18). The sensitivity of FADIBs to pulse parameters was explored by varying the pulse duration (0.2–1 s) and amplitude (ranging from −0.01 to −0.2 V and +0.01 to +0.2 V), as shown in Fig. 5c and d and Fig. S19. With increasing pulse width, the amplitude of the PSC was significantly enhanced, indicating increased ion injection into the channel. Similarly, stronger pulse amplitudes facilitated greater ionic flux through enhanced electric field strength, reflecting typical time-dependent and voltage-dependent synaptic plasticity [39]. To further explore short-term synaptic behavior, we applied two consecutive negative presynaptic pulses with a fixed short interval of 0.1 s (Fig. 5e and Fig. S20). The second EPSC response (B_2_) was notably larger than the first (B_1_), suggesting a facilitation effect caused by incomplete ion relaxation—characteristic of SRDP. To quantify this behavior, the paired-pulse facilitation (PPF) index was calculated under varying interpulse intervals. As illustrated in Fig. 5f, the PPF index exhibited a typical exponential decay with increasing interval, and the fitted curve showed strong agreement with the experimental data.

To further assess the capacity of the FADIBs for dynamic signal modulation under continuous stimulation, we applied a train of 10 successive voltage pulses and systematically varied both the pulse width and stimulation frequency. As depicted in Fig. 5g, longer pulse durations (from 0.2 to 1 s) resulted in progressively enhanced EPSC amplitudes. Figure5h demonstrates that higher stimulation frequencies (from 1 to 0.2 s per spike) led to cumulative EPSC growth and stronger synaptic responses. This behavior is a clear manifestation of SRDP, arising from insufficient ionic relaxation between closely spaced pulses. To quantify this effect, we defined the SRDP index as the ratio of the 10th EPSC amplitude to that of the 1st, 4th and 7th spikes (Fig. 5i). A pronounced increase in the SRDP index with rising stimulation frequency confirms the strong short-term memory enhancement and suitability for constructing responsive neuromorphic systems [6]. Additionally, the energy consumption of a single synaptic event was evaluated. As shown in Fig. S21, under a −100 μV stimulus, FADIBs achieved a minimum EPSC energy consumption as low as 7.5 fJ, which is markedly lower than that of most recently reported artificial synapses (Fig. 5j and Table S3) [40–47]. This can be attributed to the confined fiber architecture and dual-ion design, which facilitate straightforward ion rearrangement [48,49]. This ultra-low power operation not only enhances the feasibility of large-scale neuromorphic hardware integration but also opens up new possibilities for constructing brain-inspired systems with near-biological energy efficiency [50]. Meanwhile, as shown in Fig. S22, we carried out 100 trials of EPSC measurements in response to a 10-successive-spike voltage train (−0.1 mV, 1 s duration, 0.2 s interval). The device not only maintained stable operation throughout all cycles but also exhibited signatures of long-term memory retention.

To demonstrate the practical application of FADIBs in wearable systems, we integrated them with an electrochromic fabric to create a synaptic-controlled color-changing module. As depicted in Fig. 5k, the EPSC output of the artificial synapse gradually builds up under a sequence of presynaptic-like voltage pulses, facilitated by SRDP. This accumulated current then triggers the electrochromic response of the fabric, resulting in visible color changes. Figure 5l visually illustrates the fabric’s color transformation at various stages. Initially, the fabric maintains its original color. Upon applying multiple synaptic pulses, the rising EPSC drives the fabric to a deep green state. Once the stimulation ceases, the EPSC gradually decays; the fabric color shifts back from green but remains in a yellow-green hue for a period, sustained by the residual current. This experiment provides a compelling demonstration of the artificial synapse’s ability to effectively regulate current output, validating its potential as a control module for adaptive smart wearable systems.

## CONCLUSION

In summary, we present FADIBs that successfully integrate energy storage, ionic rectification and artificial synaptic functionalities. FADIBs are composed of a CuHCF/CNTF cathode, an Ag/CNTF anode and an NH_4_Cl/PVA gel electrolyte, where NH_4_^+^ ions undergo reversible intercalation/deintercalation at the cathode, while Cl^−^ ions participate in conversion reactions at the anode. Benefiting from the intrinsic dual-ion transport and reaction mechanism, FADIBs deliver a high energy density of 51.5 mWh cm^−3^ at a power density of 713.3 mW cm^−3^. Furthermore, the voltage-dependent asymmetric migration of NH_4_^+^ and Cl^−^ ions enables distinct current responses under forward/reverse bias, yielding an ionic rectification ratio of up to 109. By exploiting ion relaxation dynamics in the electrolyte, FADIBs emulate key neuromorphic behaviors (including EPSC/IPSC and SRDP) with ultralow energy consumption of 7.5 fJ per synaptic event. Demonstrating practical versatility, FADIBs were integrated into textile-based modules for applications spanning energy storage, harvesting and synaptic-controlled electrochromic regulation. This work not only highlights the potential of FADIBs to host multifunctionality but also establishes a foundation for developing lightweight, integrable systems in next-generation wearable electronics.

## METHODS

Details of the materials and methods are available in the Supplementary data.

## Supplementary Material

nwag062_Supplemental_File

## References

[1] Chen C, Feng J, Li J et al. Functional fiber materials to smart fiber devices. Chem Rev 2023; 123: 613–62. 10.1021/acs.chemrev.2c00192 35977344

[2] Dang C, Wang Z, Hughes-Riley T et al. Fibres—threads of intelligence—enable a new generation of wearable systems. Chem Soc Rev 2024; 53: 8790–846. 10.1039/D4CS00286E 39087714

[3] Zeng K, Shi X, Tang C et al. Design, fabrication and assembly considerations for electronic systems made of fibre devices. Nat Rev Mater 2023; 8: 552–61. 10.1038/s41578-023-00573-x

[4] Yang Y, Shao Y, Lu G et al. Fusing fibre batteries interface via biomimetic gel electrolyte. Adv Fiber Mater 2024; 6: 949–51. 10.1007/s42765-024-00448-y

[5] Xing Y, Zhou M, Si Y et al. Integrated opposite charge grafting induced ionic-junction fiber. Nat Commun 2023; 14: 2355. 10.1038/s41467-023-37884-0 37095082 PMC10126126

[6] Chen L, Ren M, Zhou J et al. Bioinspired iontronic synapse fibers for ultralow-power multiplexing neuromorphic sensorimotor textiles. Proc Natl Acad Sci USA 2024; 121: e2407971121. 10.1073/pnas.2407971121 39110725 PMC11331142

[7] Yang W, Lin S, Gong W et al. Single body-coupled fiber enables chipless textile electronics. Science 2024; 384: 74–81. 10.1126/science.adk3755 38574120

[8] Wang X, Lei P, Zheng C et al. Flexible and durable meter-long fiber-shaped Zn-ion battery enabled by zincophilic, tough double-network hydrogel electrolytes. Adv Funct Mater 2025; 35: 2500916. 10.1002/adfm.202500916

[9] Qin L, Xu C, Che Q et al. Aqueous battery fiber with high volumetric and areal power density for flexible electronics. Device 2024; 2: 100179. 10.1016/j.device.2023.100179

[10] Zhang R, Wang S, Chou S et al. Research development on aqueous ammonium-ion batteries. Adv Funct Mater 2022; 32: 2112179. 10.1002/adfm.202112179

[11] Shi M, Zhang X. Pioneering the future: principles, advances, and challenges in organic electrodes for aqueous ammonium-ion batteries. Adv Mater 2025; 37: 2415676. 10.1002/adma.202415676 39998316 PMC11962702

[12] Jiang F, Poh WC, Chen J et al. Ion rectification based on gel polymer electrolyte ionic diode. Nat Commun 2022; 13: 6669. 10.1038/s41467-022-34429-9 36335134 PMC9637189

[13] Chen X, Chen L, Zhou J et al. Self-adhesive, stretchable, and thermosensitive iontronic hydrogels for highly sensitive neuromorphic sensing-synaptic systems. Nano Lett 2024; 24: 10265–74. 10.1021/acs.nanolett.4c02614 39116304

[14] Chouhdry HH, Lee DH, Bag A et al. A flexible artificial chemosensory neuronal synapse based on chemoreceptive ionogel-gated electrochemical transistor. Nat Commun 2023; 14: 821. 10.1038/s41467-023-36480-6 36788242 PMC9929093

[15] Han L, Ling Y, Gong W et al. In-situ dynamic compensation strategy enables Jahn-Teller effect suppression in Zn-doped dual-active-site copper hexacyanoferrate cathodes for high-energy and ultra-stable ammonium-ion storage. Angew Chem Int Ed 2025; 64: e202507427. 10.1002/anie.202507427 40405539

[16] Liang G, Mo F, Wang D et al. Commencing mild Ag–Zn batteries with long-term stability and ultra-flat voltage platform. Energy Storage Mater 2020; 25: 86–92. 10.1016/j.ensm.2019.10.028

[17] Ling Y, He B, Han L et al. Two-electron redox chemistry enables potassium-free copper hexacyanoferrate as high-capacity cathode for aqueous Mg-ion battery. InfoMat 2024; 6: e12549. 10.1002/inf2.12549

[18] Guan Q, Li Y, Bi X et al. Dendrite-free flexible fiber-shaped Zn battery with long cycle life in water and air. Adv Energy Mater 2019; 9: 1901434. 10.1002/aenm.201901434

[19] He B, Zhou Z, Man P et al. V_2_O_5_ nanosheets supported on 3D N-doped carbon nanowall arrays as an advanced cathode for high energy and high power fiber-shaped zinc-ion batteries. J Mater Chem A 2019; 7: 12979–86. 10.1039/C9TA01164A

[20] Liu C, Li Q, Cao J et al. Superstructured α-Fe_2_O_3_ nanorods as novel binder-free anodes for high-performing fiber-shaped Ni/Fe battery. Sci Bull 2020; 65: 812–9. 10.1016/j.scib.2020.03.004 36659199

[21] Liu Y, Wang J, Zeng Y et al. Interfacial engineering coupled valence tuning of MoO_3_ cathode for high-capacity and high-rate fiber-shaped zinc-ion batteries. Small 2020; 16: 1907458. 10.1002/smll.201907458 32068969

[22] Xiong T, He B, Zhou T et al. Stretchable fiber-shaped aqueous aluminum ion batteries. EcoMat 2022; 4: e12218. 10.1002/eom2.12218

[23] Yu J, Cai D, Si J et al. MOF-derived NiCo_2_S_4_ and carbon hybrid hollow spheres compactly concatenated by electrospun carbon nanofibers as self-standing electrodes for aqueous alkaline Zn batteries. J Mater Chem A 2022; 10: 4100–9. 10.1039/D1TA10597C

[24] Zhang Q, Zhou Z, Pan Z et al. All-metal-organic framework-derived battery materials on carbon nanotube fibers for wearable energy-storage device. Adv Sci 2018; 5: 1801462. 10.1002/advs.201801462 PMC6299715 30581717

[25] Zhang X, Pei Z, Wang C et al. Flexible zinc-ion hybrid fiber capacitors with ultrahigh energy density and long cycling life for wearable electronics. Small 2019; 15: 1903817. 10.1002/smll.201903817 31609075

[26] Zhang E, Fulik N, Hao GP et al. An asymmetric supercapacitor-diode (CAPode) for unidirectional energy storage. Angew Chem Int Ed 2019; 58: 13060–5. 10.1002/anie.201904888 PMC6772186 31283103

[27] Feng J, Wang Y, Xu Y et al. Construction of supercapacitor-based ionic diodes with adjustable bias directions by using poly(ionic liquid) electrolytes. Adv Mater 2021; 33: 2100887. 10.1002/adma.202100887 34165843

[28] Tang P, Tan W, Li F et al. A pseudocapacitor diode based on ion-selective surface redox effect. Adv Mater 2023; 35: 2209186. 10.1002/adma.202209186 36564639

[29] Bahrawy A, Galek P, Gellrich C et al. A gated highly variable pseudocapacitor based on redox-window control (G-CAPode). Energy Storage Mater 2025; 74: 103872. 10.1016/j.ensm.2024.103872

[30] Zhou H, Li P, Zhang E et al. General design concepts for CAPodes as ionologic devices. Angew Chem Int Ed 2023; 62: e202305397. 10.1002/anie.202305397 37394690

[31] Gellrich C, Shupletsov L, Galek P et al. A precursor-derived ultramicroporous carbon for printing iontronic logic gates and super-varactors. Adv Mater 2024; 36: 2401336. 10.1002/adma.202401336 38700498

[32] Ma Y, Tang P, Miao Z et al. Flexible planar micro supercapacitor diode. J Energy Chem 2024; 93: 429–35. 10.1016/j.jechem.2024.02.008

[33] Bahrawy A, Galek P, Gellrich C et al. Advanced redox electrochemical capacitor diode (CAPode) based on parkerite (Ni_3_Bi_2_S_2_) with high rectification ratio for iontronic applications. Adv Funct Mater 2024; 34: 2405640. 10.1002/adfm.202405640

[34] Tang P, Jing P, Luo Z et al. Modulating ionic hysteresis to selective interaction mechanism toward transition from supercapacitor-memristor to supercapacitor-diode. Nano Lett 2025; 25: 5415–24. 10.1021/acs.nanolett.5c00596 40111392

[35] Zhao GX, Pan ZT, Xu Y et al. Unidirectional bias study based on nickel foam electrochemical ion diode. Adv Funct Mater 2025; 35: 2417394. 10.1002/adfm.202417394

[36] Zhang Y, Tan CMJ, Toepfer CN et al. Microscale droplet assembly enables biocompatible multifunctional modular iontronics. Science 2024; 386: 1024–30. 10.1126/science.adr0428 39607936

[37] Wang L, Wang S, Xu G et al. Ionic potential relaxation effect in a hydrogel enabling synapse-like information processing. ACS Nano 2024; 18: 29704–14. 10.1021/acsnano.4c09154 39412087

[38] Xiong T, Li C, He X et al. Neuromorphic functions with a polyelectrolyte-confined fluidic memristor. Science 2023; 379: 156–61. 10.1126/science.adc9150 36634194

[39] Chen L, Li R, Yuan S et al. Fiber-shaped artificial optoelectronic synapses for wearable visual-memory systems. Matter 2023; 6: 925–39. 10.1016/j.matt.2022.12.001

[40] Assi DS, Huang H, Karthikeyan V et al. Quantum topological neuristors for advanced neuromorphic intelligent systems. Adv Sci 2023; 10: 2300791. 10.1002/advs.202300791 PMC10460853 37340871

[41] de Boer JJ, Ehrler B. Scalable microscale artificial synapses of lead halide perovskite with femtojoule energy consumption. ACS Energy Lett 2024; 9: 5787–94. 10.1021/acsenergylett.4c02360 39698340 PMC11650764

[42] He J, Wei R, Ge S et al. Artificial visual-tactile perception array for enhanced memory and neuromorphic computations. InfoMat 2024; 6: e12493. 10.1002/inf2.12493

[43] Luo Z, Xie Y, Li Z et al. Plasmonically engineered light-matter interactions in Au-nanoparticle/MoS_2_ heterostructures for artificial optoelectronic synapse. Nano Res 2022; 15: 3539–47. 10.1007/s12274-021-3875-0

[44] Tang J, He C, Tang J et al. A reliable all-2D materials artificial synapse for high energy-efficient neuromorphic computing. Adv Funct Mater 2021; 31: 2011083. 10.1002/adfm.202011083

[45] Wang X, Yan Y, Li E et al. Stretchable synaptic transistors with tunable synaptic behavior. Nano Energy 2020; 75: 104952. 10.1016/j.nanoen.2020.104952

[46] Xie Z, Zhuge C, Zhao Y et al. All-solid-state vertical three-terminal N-type organic synaptic devices for neuromorphic computing. Adv Funct Mater 2022; 32: 2107314. 10.1002/adfm.202107314

[47] Yu T, Zhao Z, Jiang H et al. MoTe_2_-based low energy consumption artificial synapse for neuromorphic behavior and decimal arithmetic. Mater Today Chem 2023; 27: 101268. 10.1016/j.mtchem.2022.101268

[48] Huang M, Schwacke M, Onen M et al. Electrochemical ionic synapses: progress and perspectives. Adv Mater 2023; 35: 2205169. 10.1002/adma.202205169 36300807

[49] Talin AA, Meyer J, Li J et al. Electrochemical random-access memory: progress, perspectives, and opportunities. Chem Rev 2025; 125: 1962–2008. 10.1021/acs.chemrev.4c00512 39960411

[50] Lee Y, Park H-L, Kim Y et al. Organic electronic synapses with low energy consumption. Joule 2021; 5: 794–810. 10.1016/j.joule.2021.01.005

