# Supplementary material for: Fiber-shaped aqueous dual-ion batteries integrating rectification and synaptic functions
Source: Natl Sci Rev. 2026 Jan 28;13(6):nwag062. doi: 10.1093/nsr/nwag062 (PMC12988485; doi:10.1093/nsr/nwag062)
Supplement: nwag062_Supplemental_File [file nwag062_supplemental_file.pdf]

SUPPLEMENTARY DATA

Fiber-Shaped Aqueous Dual-Ion Batteries Integrating Rectification and Synaptic Functions

Siyuan Ye[1,2,3†], Lijie Han[1†], Yu Meng[1], Long Chen[4], Yaowu Li[1], Shan Cong[1], Guan Wu[2] and Qichong Zhang[1,3]*

[1]Key Laboratory of Multifunctional Nanomaterials and Smart Systems, Suzhou Institute of Nano-Tech and Nano-Bionics, Chinese Academy of Sciences, Suzhou 215123, China

[2]National Engineering Lab for Textile Fiber Materials and Processing Technology, Zhejiang Sci-Tech University, Hangzhou 310018, China

[3]Jiangsu Key Laboratory of Organoid Engineering and Precision Medicine, Suzhou Institute of Nano-Tech and Nano-Bionics, Chinese Academy of Sciences, Suzhou 215123, China

[4]School of Electrical and Electronic Engineering, Nanyang Technological University, Singapore 639798, Singapore

[†]These authors contributed equally to this work.

***Corresponding authors.** E-mail: qczhang2016@sinano.ac.cn

Experimental Section

Synthesis of Cu/CNTF

Cu/CNTF was prepared via in situ electrochemical deposition using a three-electrode setup, with CNTF, Pt wire, and Ag/AgCl as the working, counter, and reference electrodes, respectively. The electrolyte consisted of 180 g L^{-1} $CuSO_4$, 33 mL concentrated H_2SO_4, and 80 mL concentrated HCl in 1 L deionized water. Deposition was performed at a constant current density of 0.05 A cm^{-2}. Afterward, the electrode was rinsed with deionized water and dried under vacuum at 60 °C for 12 h.

Synthesis of CuO/CNTF

CuO/CNTF was obtained by immersing Cu/CNTF in an alkaline oxidation solution composed of 3.2 g NaOH and 0.1 g $(NH_4)_2S_2O_8$ dissolved in 30 mL DI water. The reaction proceeded at room temperature for 24 h. The resulting electrode was washed thoroughly with DI water and dried under vacuum at 60 °C for 12 h.

Synthesis of CuHCF/CNTF

For CuHCF deposition, 0.5 g $K_3[Fe(CN)_6]$ and 0.1 g $C_6H_3(COOH)_3$ were dissolved separately in 20 mL DI water (solution A) and 10 mL ethanol (solution B). Solutions A and B were mixed and stirred for 10 min to form solution C. CuO/CNTF was immersed in solution C for 24 h, followed by rinsing with DI water and vacuum drying at 60 °C for 12 h to obtain CuHCF/CNTF.

Synthesis of Ag/CNTF

Ag/CNTF was fabricated via electrochemical deposition using a two-electrode system. The electrolyte was prepared by dissolving 1.7 g $AgNO_3$, 0.031 mL HNO_3, and 0.144 g $C_6H_8O_7$ in 50 mL deionized water, forming a clear and homogeneous solution. A CNTF was used as the

working electrode, and a Pt wire served as the counter electrode. Deposition was performed at a constant current density of −0.02 A cm^{-2}. The resulting Ag/CNTF composite was rinsed and dried for subsequent use.

Fabrication of FADIBs

CuHCF/CNTF and Ag/CNTF were used as the cathode and anode electrodes, respectively. A uniform layer of PVA/NH_4Cl gel electrolyte was applied between them to ensure good contact. The assembly was dried at 60 °C for 2 min, and this step was repeated until the gel reached a uniform and suitable thickness. The two fiber electrodes were then twisted together at an appropriate angle to increase interfacial contact. After the gel fully solidified, the device was encapsulated to prevent water evaporation and electrolyte leakage. The resulting fiber-based device was used for electrochemical measurements.

Characterizations

Morphologies of the samples were characterized using a scanning electron microscope (SEM) (Hitachi S-4800, 5 kV). The microstructure and high-resolution transmission electron microscope (TEM) images were acquired through an FEI Tecnai G2 20 high-resolution transmission electron microscope at an acceleration voltage of 200 kV. Wide Angle X-ray Scattering (WAXS) experiments were performed using a Xenocs Xeuss 3.0 laboratory beamline at the Vacuum Interconnected Nanotech Workstation (Nano-X) of Suzhou Institute of Nano-Tech and Nano-Bionics, Chinese Academy of Sciences. An X-ray wavelength of λ=1.54189 Å and a beam size of (0.9×0.9) mm^2 was employed for the measurements. The scattered intensities were collected using a Dectris EIGER2 R 1M detector with a sample to detector distance of 60 mm, with an instrument resolution of ~0.06 $Å^{-1}$. X-ray photoelectron spectroscopy (XPS) measurements were performed at Nano-X using a spectrometer

(ESCALAB Xi$^+$, Thermo Scientific) equipped with a microfocused monochromatic Al Kα X-ray source. Raman spectra were recorded in invia Qontor (Renishaw) with a laser light source of 532 nm.

Electrical and Performance Measurements

Electrochemical measurements were performed using an electrochemical workstation (CHI 760E, Chenhua and CS3140, Corrtest) at room temperature. The electrochemical properties of the single electrodes were measured using a three-electrode system in 1 M NH_4Cl aqueous electrolyte, with the cathode materials serving as the working electrodes, a Pt wire as the counter electrode, and an Ag/AgCl electrode as the reference electrode. Electrochemical properties of the FADIBs were measured in the NH_4Cl/PVA electrolyte. Rectification and synaptic performance of the FADIBs were tested using a Keithley 2450B source meter, connected to a probe station under ambient conditions.

Fabrication of Triboelectric Harvesting Module

Nylon fabric and PDMS film with dimensions of 10×20 cm was selected as the positive and negative triboelectric layers, respectively, with copper serving as the electrodes. The triboelectric generator was operated under a contact force of 10 N and a contact frequency of 1 Hz. It was connected to a rectifier bridge composed of four FADIBs, as well as a 3.3μf capacitor component. The voltage data of the capacitor component were collected using a Keithley 6517 high-resistance/low-current electrometer.

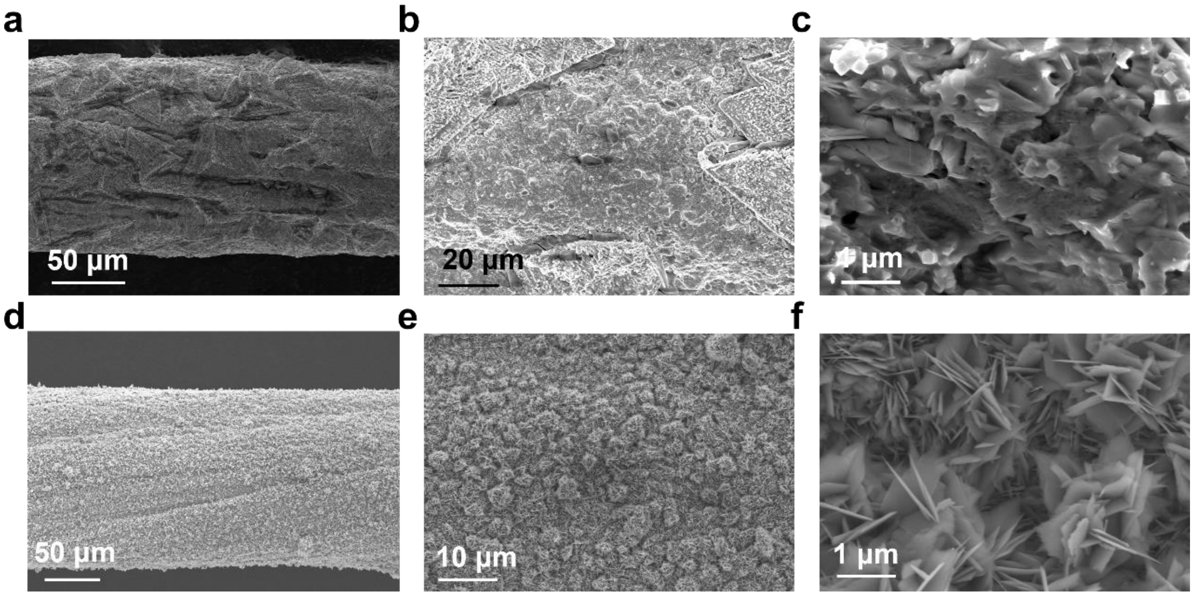

Fig S1. (a-c) SEM images of Cu/CNTF. (d-f) SEM images of CuO/CNTF.

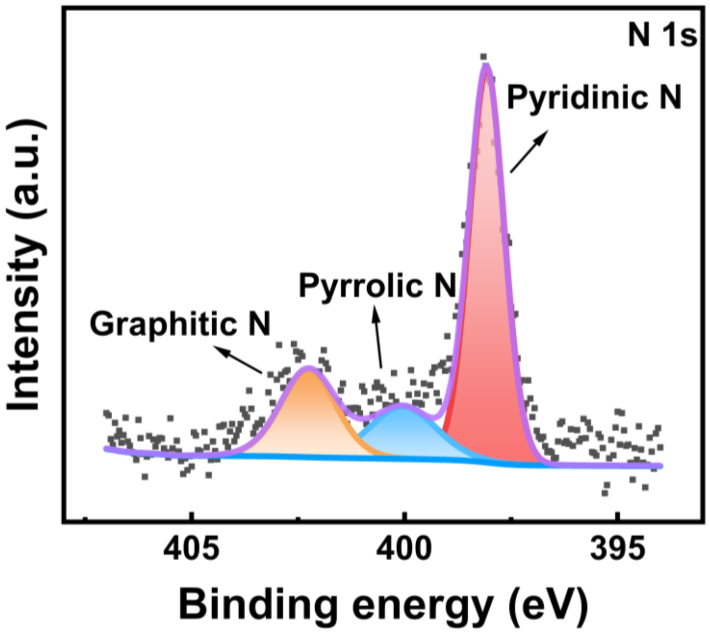

Fig S2. N 1s XPS spectra of CuHCF/CNTF.

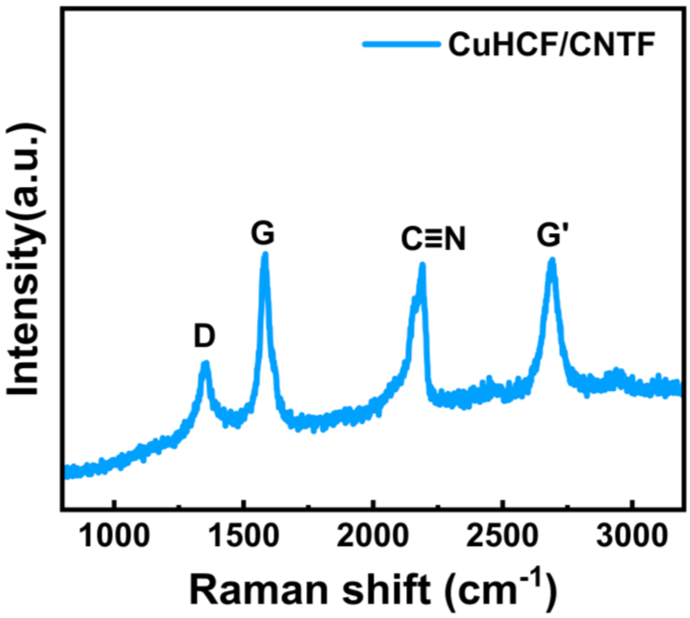

Fig S3. Raman spectra of CuHCF/CNTF.

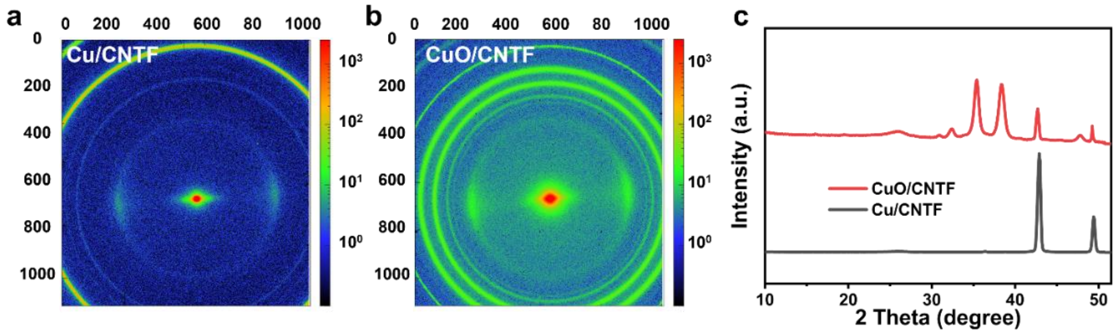

Fig S4. 2D WAXS pattern collected from (a) Cu/CNTF and (b) CuO/CNTF. (c) 1D WAXS curves of Cu/CNTF and CuO/CNTF.

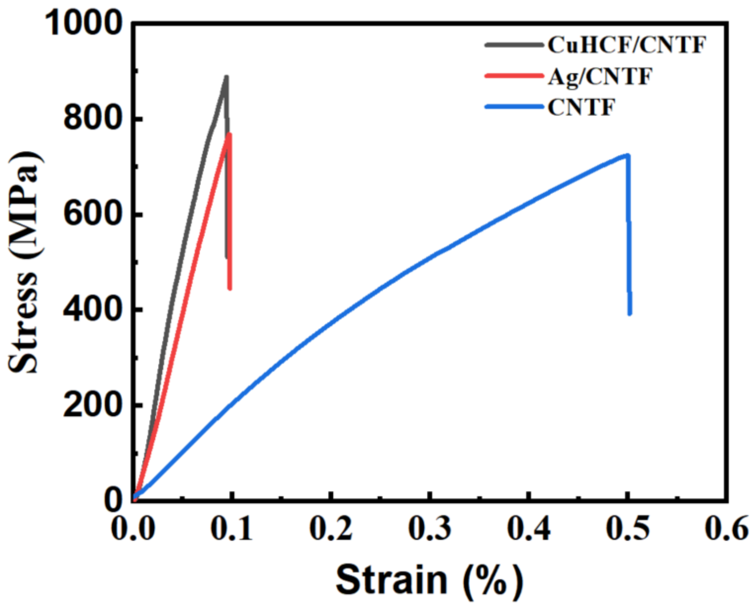

Fig S5. Tensile stress-strain curves of CuHCF/CNTF, Ag/CNTF, and CNTF.

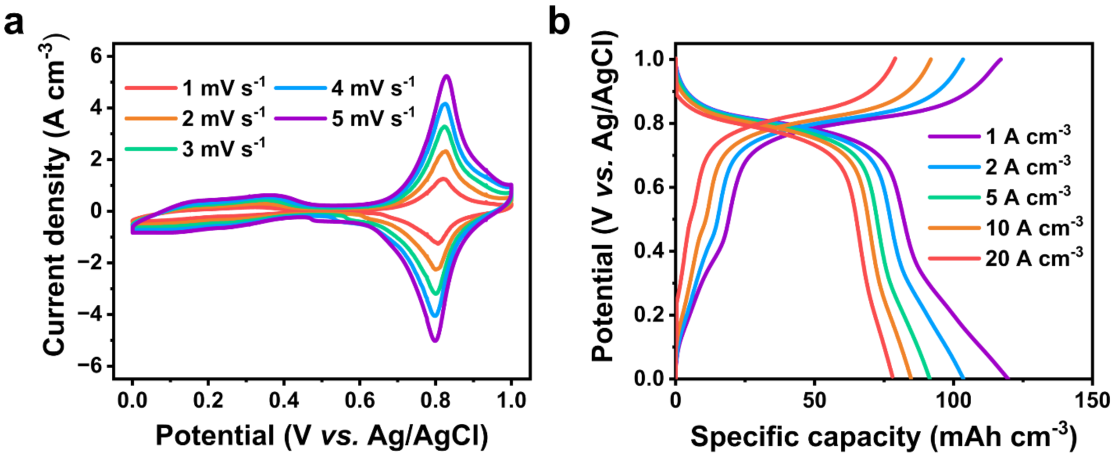

Fig S6. (a) CV curves of CuHCF/CNTF at different scan rates. (b) GCD curves of CuHCF/CNTF at different current densities.

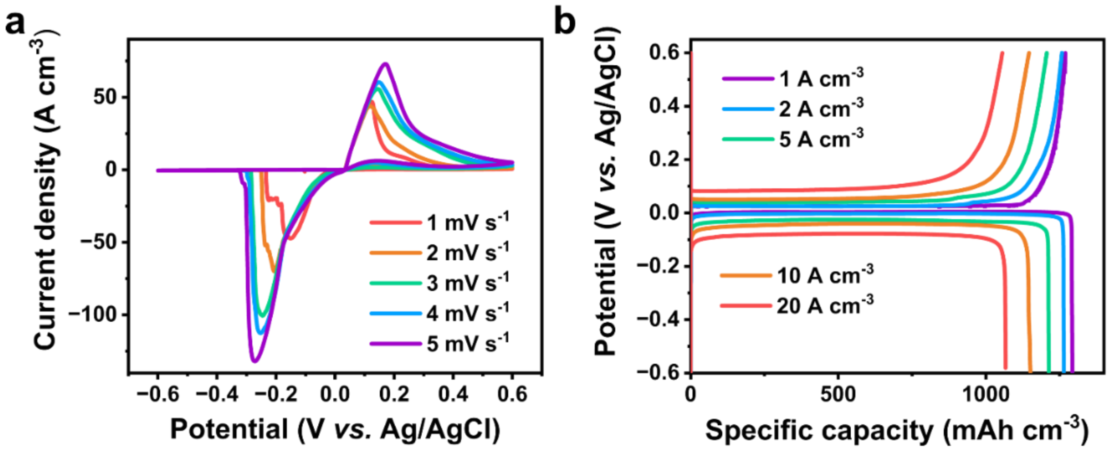

Fig S7. (a) CV curves of Ag/CNTF at different scan rates. (b) GCD curves of Ag/CNTF at different current densities.

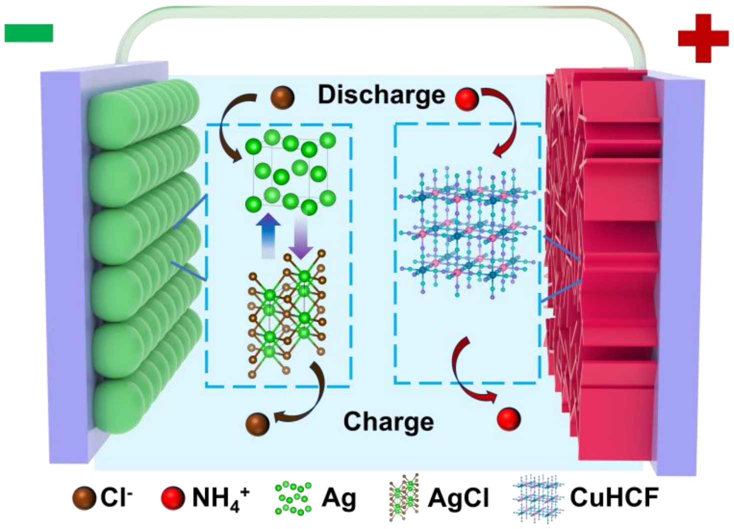

Fig S8. Schematic of the energy storage mechanism of FADIBs.

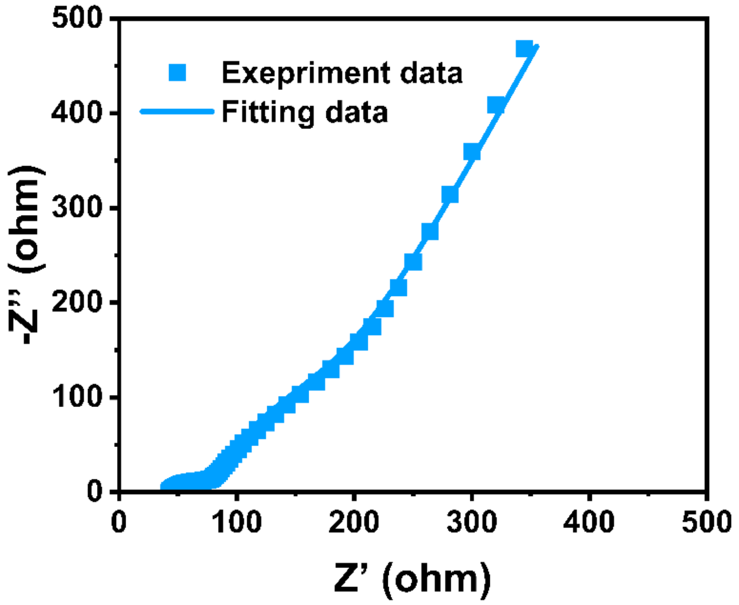

Fig S9. Nyquist plots of FADIBs.

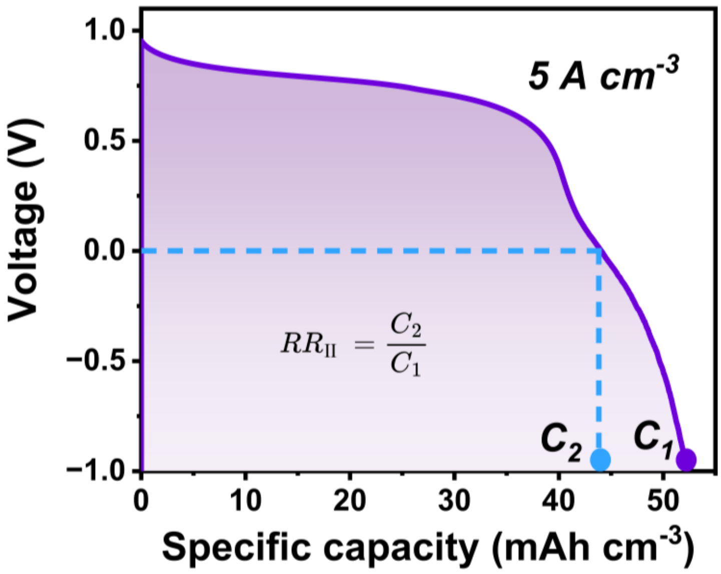

Fig S10. Schematic diagram of the calculation method for RR_{II}.

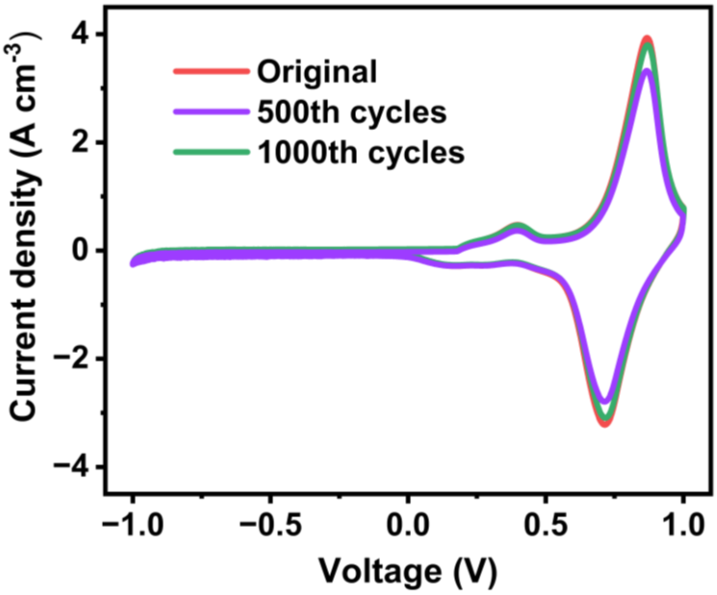

Fig S11. CV curves of the FADIBs at the initial cycle, the 500th cycle and the 1000th cycle.

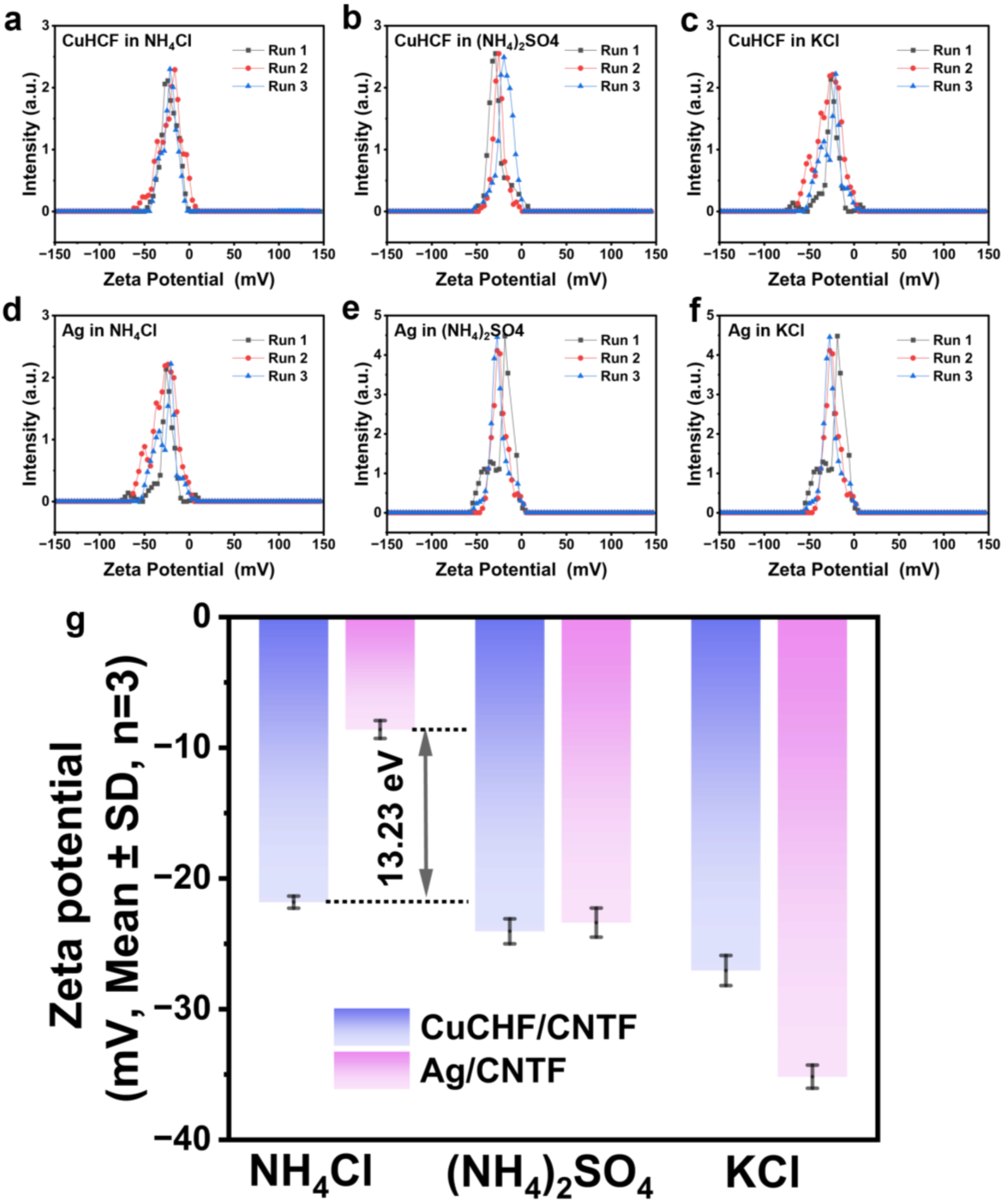

Fig S12. Zeta potential of CuHCF/CNTF and Ag/CNTF in 0.01 M NH_4Cl, $(NH_4)_2SO_4$, KCl electrolyte.

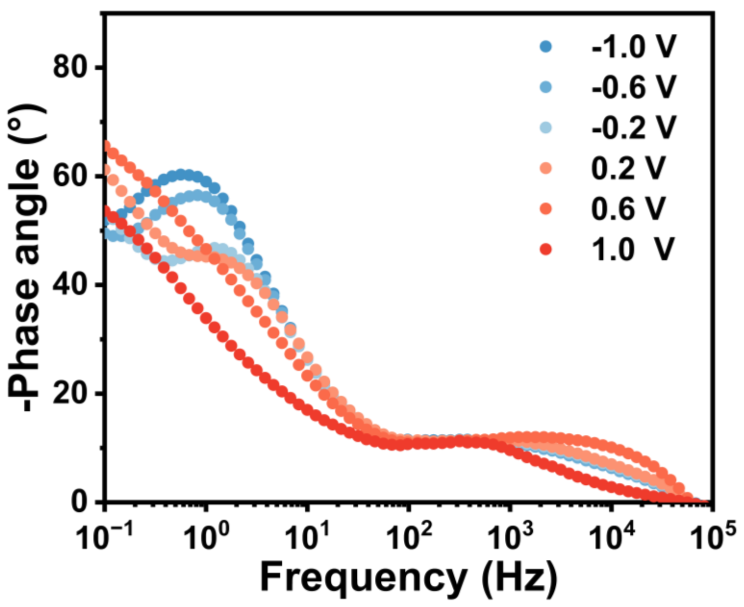

Fig S13. Bode plots of the FADIBs under DC biases.

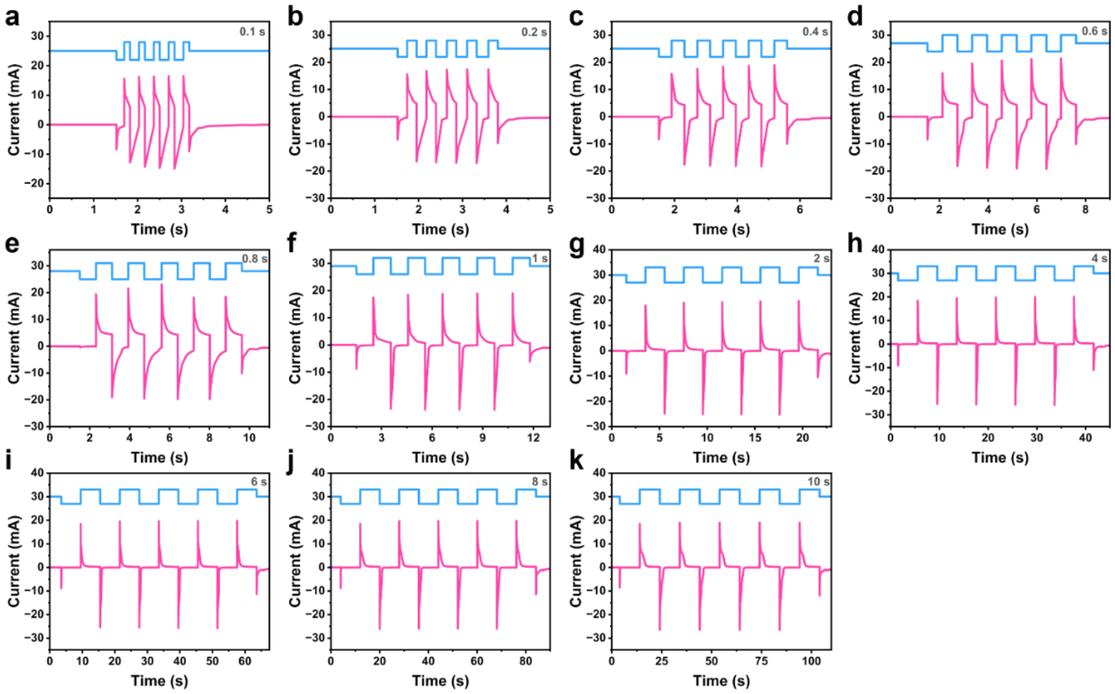

Fig S14. Real-time current response of the FADIBs at a repetitive −1 V and +1 V.

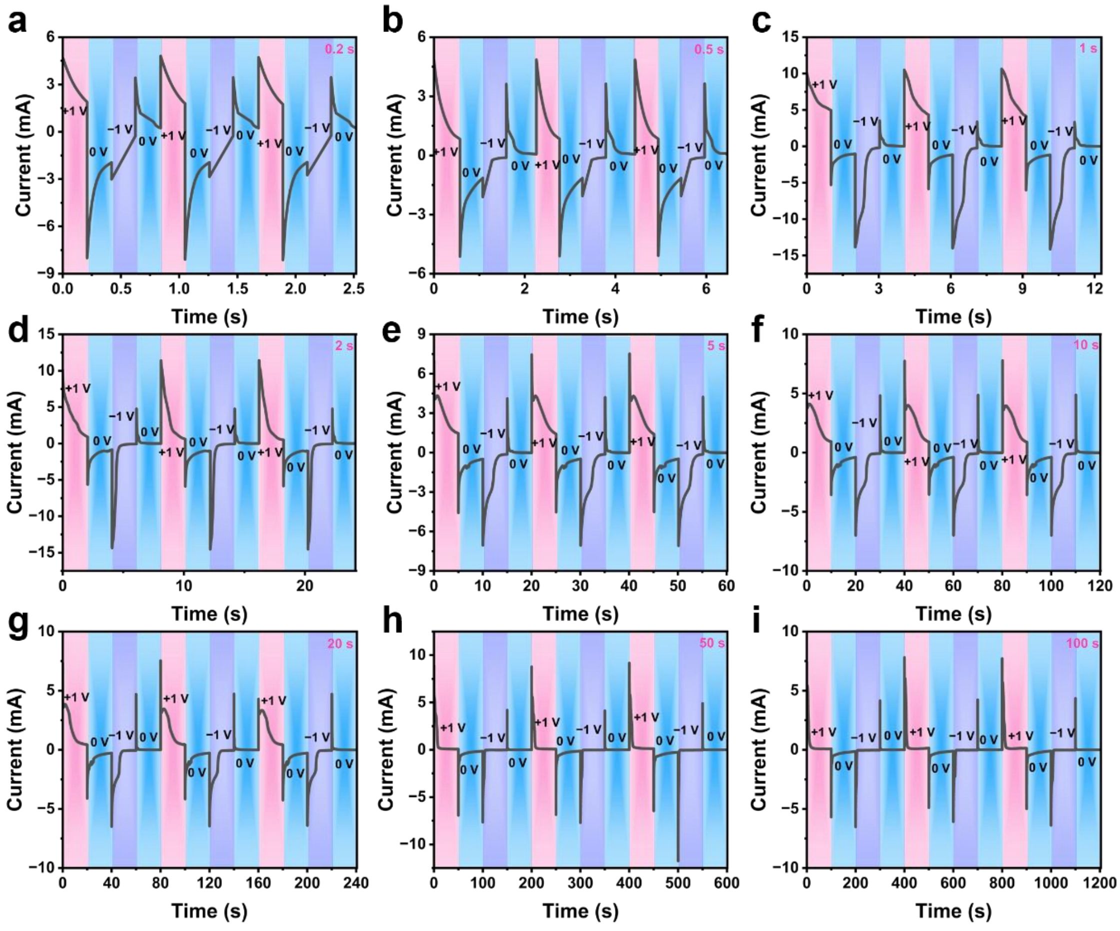

Fig S15 The I−t curves for an external bias alternating between +1 V and −1 V at different durations.

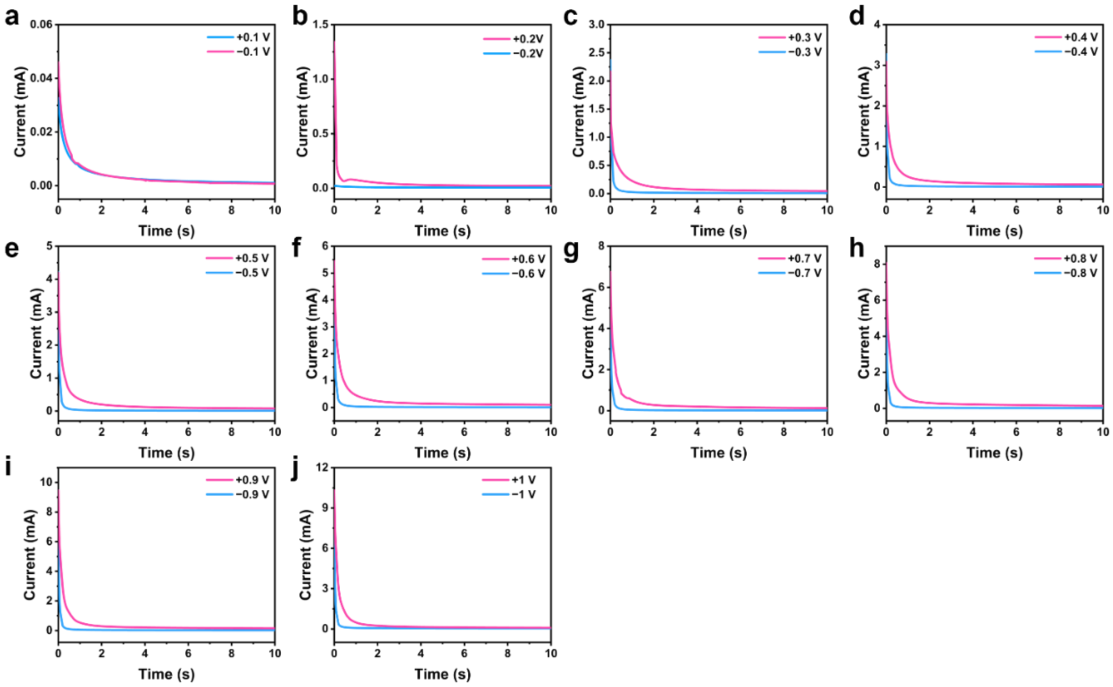

Fig S16. Current variation of the FADIBs under forward and reverse biases at different voltages for 10 s at each voltage.

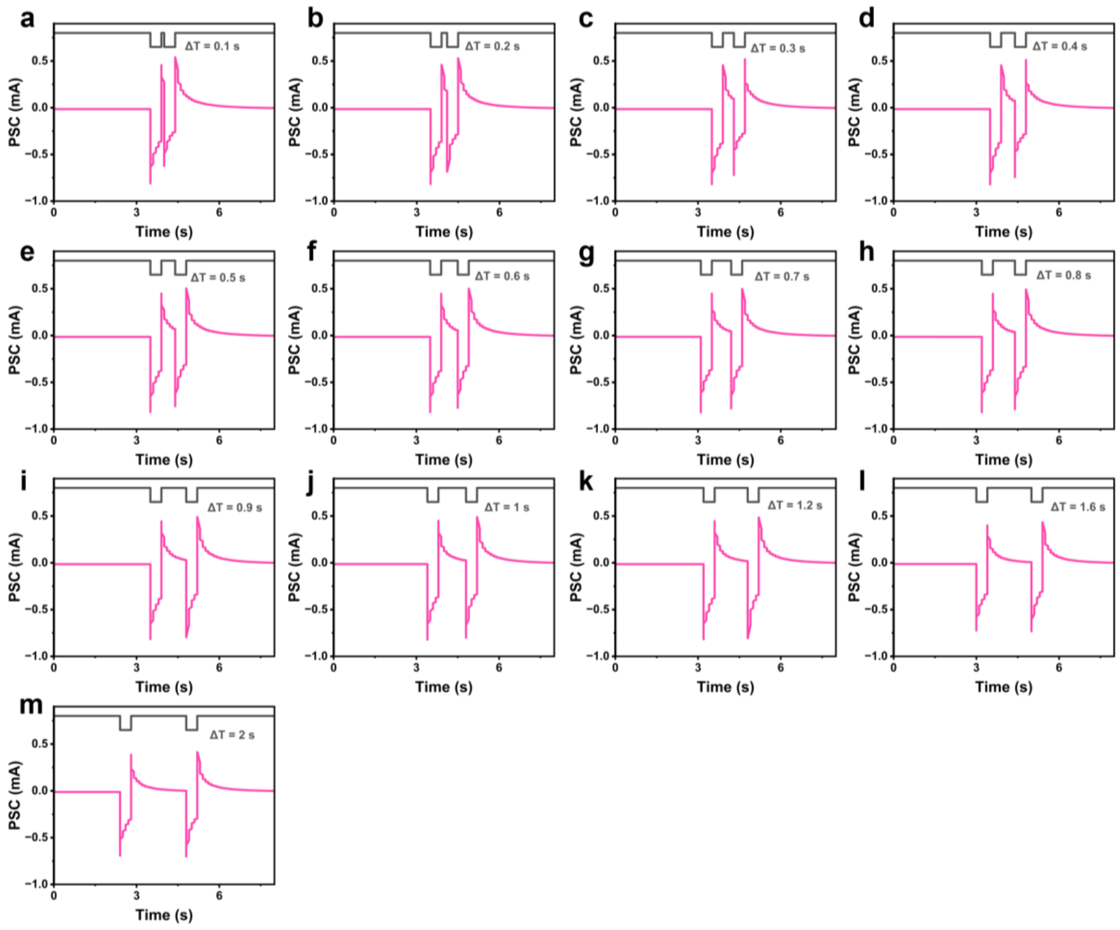

Fig S17. Pulse facilitation behavior of the FADIBs (V = −0.1 V) at different intervals (pulse duration = 0.4 s).

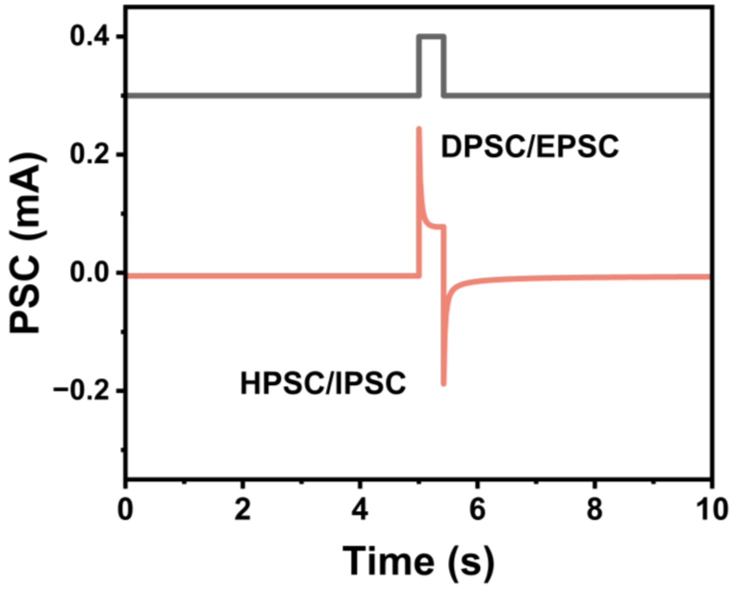

Fig S18. PSC of EPSC/IPSC and DPSC/HPSC activated by a positive presynaptic pulse.

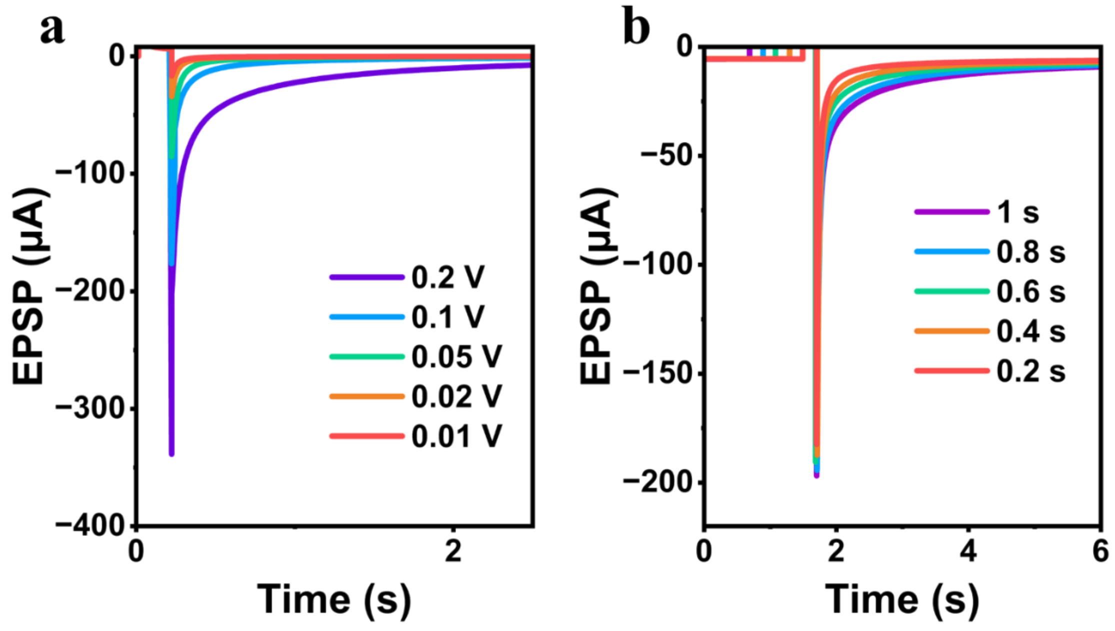

Fig S19. Spike-dependent plasticity achieved by changing (a) spike duration and (b) spike intensity.

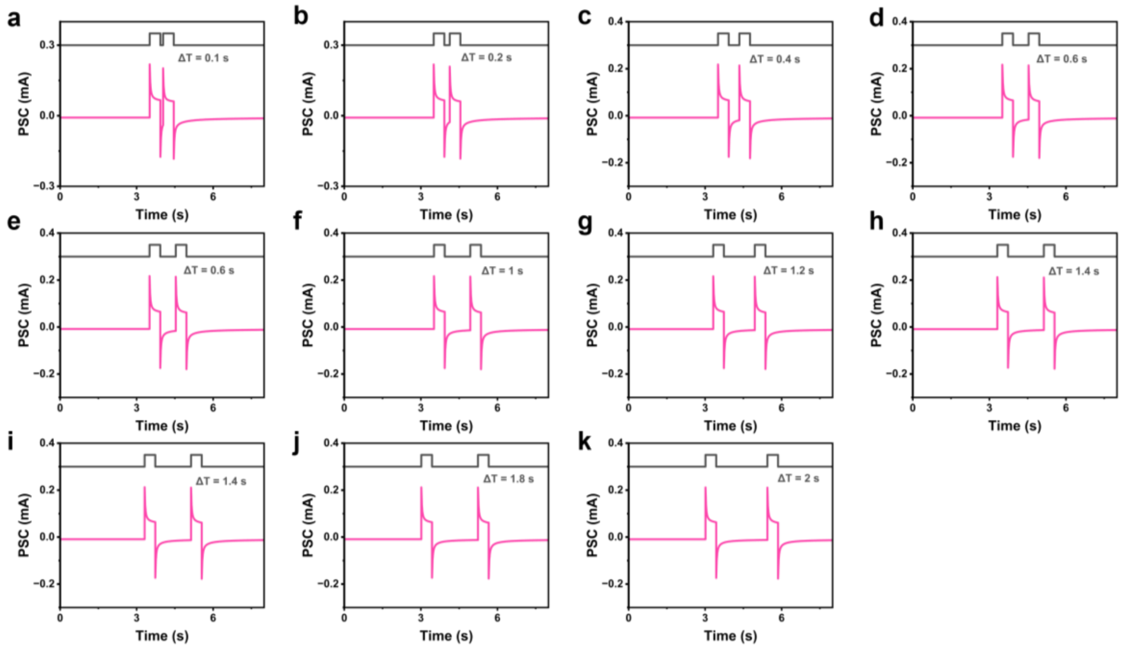

Fig S20. Pulse facilitation behavior of the FADIBs (V = +0.1 V) at different intervals (pulse duration = 0.4 s).

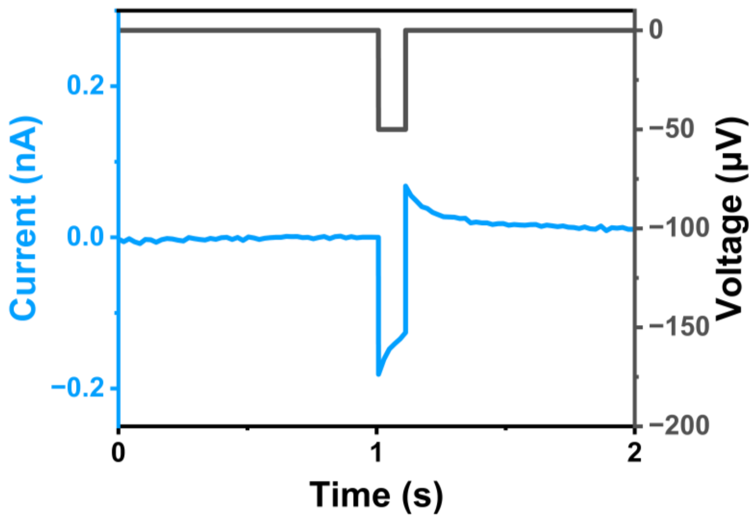

Fig S21. The minimum driving voltage stimulation of the FADIBs.

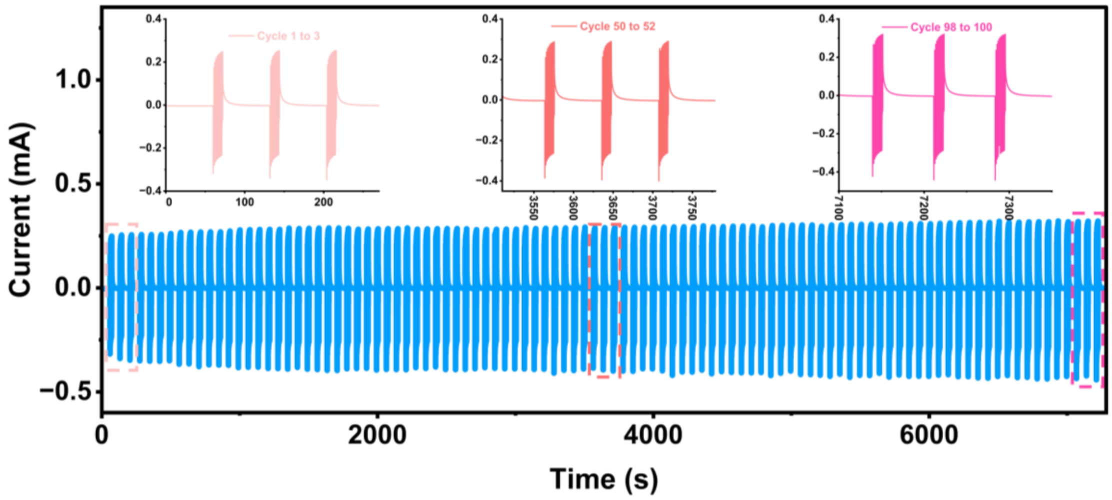

Fig S22. EPSC response to 10 consecutive spikes (V = −100 mV, duration = 1 s, spike interval = 0.2 s) across 100 trials.

Table S1. Comparison of FADIBs and other fiber-shaped batteries in energy density, power density, and electrolyte.

Fiber-shaped Solid-state Battery Devices	Power Density (mWh cm^{-3})	Energy Density (mWh cm^{-3})	Electrolytes	References
ZFCF//CNWA	73.7	5.63	KOH and $Zn(Ac)_2$	[1]
CNTF@NC@V_2O_5//Zn	56	40.8	$ZnCl_2$/PVA	[2]
MnHCF//GO/MoO3	99	30.6	PVA/$Al(CF_3SO_3)_3$	[3]
α-Fe_2O_3@PPy NRs/CNTF//$CoNiO_2$@$Ni(OH)_2$ NWAs/CNTF	228.2	15.47	KOH/PVA	[4]
$NiCo_2S_4$/HCS//Zn	265	43.9	KOH/PVA	[5]
S-α-Fe_2O_3/OCNTF//NiZnCoP @CNTF	300	30.61	KOH/PVA	[6]
rGO/CNT//Zn/C	179.9	48.5	$ZnSO_4$/PVA	[7]
PMoAl//Zn	43	14.4	ZnCl/PVA	[8]
CuHCF/CNTF//Ag/CNTF	713.3	51.5	NH_4Cl/PVA	This work

Table S2. Comparison of FADIBs and other CAPodes in electrolyte, RR_I, RR_{II}, and voltage windows.

Devieces	Electrolytes	RR_I	RR_{II}	Voltage Windows	References
C_{micro}//C_{meso}	1M $TBABF_4$	12	90%	−2-2 V	[9]
YP-50F carbon//YP-50F carbon	Poly(ionic liquid)s	~9	~80%	−1-1 V	[10]
$ZnCo_2O_4$//YP-50F carbon	1 M KOH	14.6	89%	−1.2-1.2 V	[11]
$Ni_3Bi_2S_2$@Ni//AC@Ni	1 M KOH	34	90%	−2-2 V	[12]
C_{micro}//C_{meso}	1 M $TPABF_4$(CAN)/20 %EmimPAF(ACN)	14/ 10	90%/ 80%	−2-2 V	[13]
C_{micro}//C_{meso}	1 M $TBABF_4$	10.3	82.4%	−2-2 V	[14]
$ZnCo_2O_4$//YP-50F	1 M KOH/PVA	11.9	80%	−1.2-1.2 V	[15]
$Ni_3Bi_2S_2$@Ni//AC@Ni	1 M KOH	37	96%	−0.7-0.7 V	[16]
ZIF-7//YP-50F	1 M KOH	9.9	92%	−1-1.5 V	[17]
Nickel foam//YP-50F	1 M KOH	5.41	85%	−1-1 V	[18]
CuHCF/CNTF//Ag/CNTF	1 M NH_4Cl/PVA	109	94.85%	−1-1 V	This work

Table S3. Comparison of FADIBs and other artificial synaptic device in energy consumption per synaptic event.

Years	Devices	Energy Consumption Per Synaptic Event	References
2020	Ag/ITO/PDMS	0.9 nJ	[19]
2020	PDMS-CNTs/P3HT-NF	96.6 nJ	[20]
2021	MoS_2/FLG/PDMS	18 fJ	[21]
2021	Au-NP/MoS_2/(SiO_2/Si)	34.7 pJ	[22]
2022	(PEO/$LiClO_4$)/NDI-gTVT	6.16 pJ	[23]
2022	Ag/$MoTe_2$/ITO	74.3 pJ	[24]
2023	Ag/$SnSe_{1-x}Te_x$/FTO	10 pJ	[25]
2024	Pt/(TaO_x/TiO_y)/Ti/(SiO_2/Si)	12.69 nJ	[26]
2025	(CuHCF/CNTF)/(NH_4Cl/PVA)/(Ag/CNTF)	7.5 fJ	This work

REFERENCES

1. Guan Q, Li Y, Bi X *et al.* Dendrite-free flexible fiber-shaped Zn battery with long cycle life in water and air. *Adv Energy Mater*. 2019; **9**: 1901434.

2. He B, Zhou Z, Man P *et al.* V_2O_5 nanosheets supported on 3D N-doped carbon nanowall arrays as an advanced cathode for high energy and high power fiber-shaped zinc-ion batteries. *J Mater Chem A*. 2019; **7**: 12979-12986.

3. Xiong T, He B, Zhou T *et al.* Stretchable fiber-shaped aqueous aluminum ion batteries. *EcoMat*. 2022; **4**: e12218.

4. Liu C, Li Q, Cao J *et al.* Superstructured α-Fe_2O_3 nanorods as novel binder-free anodes for high-performing fiber-shaped Ni/Fe battery. *Sci Bull*. 2020; **65**: 812-819.

5. Yu J, Cai D, Si J *et al.* MOF-derived $NiCo_2S_4$ and carbon hybrid hollow spheres compactly concatenated by electrospun carbon nanofibers as self-standing electrodes for aqueous alkaline Zn batteries. *J Mater Chem A*. 2022; **10**: 4100-4109.

6. Zhang Q, Zhou Z, Pan Z *et al.* All-metal-organic framework-derived battery materials on carbon nanotube fibers for wearable energy-storage device. *Adv Sci*. 2018; **5**: 1801462

7. Zhang X, Pei Z, Wang C *et al.* Flexible zinc-ion hybrid fiber capacitors with ultrahigh energy density and long cycling life for wearable electronics. *Small*. 2019; **15**: 1903817.

8. Liu Y, Wang J, Zeng Y *et al.* Interfacial engineering coupled valence tuning of MoO_3 cathode for high-capacity and high-rate fiber-shaped Zinc-ion batteries. *Small*. 2020; **16**: 1907458.

9. Zhang E, Fulik N, Hao GP *et al.* An asymmetric supercapacitor-diode (CAPode) for unidirectional energy storage. *Angew Chem Int Ed*. 2019; **58**: 13060-13065.

10. Feng J, Wang Y, Xu Y *et al.* Construction of supercapacitor-based ionic diodes with adjustable bias directions by using poly(ionic liquid) electrolytes. *Adv Mater*. 2021; **33**: 2100887.

11. Tang P, Tan W, Li F *et al.* A pseudocapacitor diode based on ion-selective surface redox effect. *Adv Mater*. 2023; **35**: 2209186.

12. Bahrawy A, Galek P, Gellrich C *et al.* A gated highly variable pseudocapacitor based on redox-window control (G-CAPode). *Energy Storage Mater*. 2025; **74**: 103872.

13. Zhou H, Li P, Zhang E *et al.* General design concepts for CAPodes as ionologic devices. *Angew Chem Int Ed*. 2023; **62**: e202305397.

14. Gellrich C, Shupletsov L, Galek P *et al.* A precursor-derived ultramicroporous carbon for printing iontronic logic gates and super-varactors. *Adv Mater*. 2024; **36**: 2401336.

15. Ma Y, Tang P, Miao Z *et al.* Flexible planar micro supercapacitor diode. *J Energy Chem*. 2024; **93**: 429-435.

16. Bahrawy A, Galek P, Gellrich C *et al.* Advanced redox electrochemical capacitor diode (CAPode) based on parkerite ($Ni_3Bi_2S_2$) with High rectification ratio for iontronic applications. *Adv Funct Mater*. 2024; **34**: 2405640.

17. Tang P, Jing P, Luo Z *et al.* Modulating ionic hysteresis to selective interaction mechanism toward transition from supercapacitor-memristor to supercapacitor-diode. *Nano Lett*. 2025; **25**: 5415-5424.

18. Zhao GX, Pan ZT, Xu Y *et al.* Unidirectional bias study based on nickel foam electrochemical ion diode. *Adv Funct Mater*. 2025; **35**: 2417394.

19. Yu F, Cai JC, Zhu LQ *et al.* Artificial tactile perceptual neuron with nociceptive and pressure decoding abilities. *ACS Appl Mater Interfaces*. 2020; **12**: 26258-26266.

20. Wang X, Yan Y, Li E *et al.* Stretchable synaptic transistors with tunable synaptic behavior. *Nano Energy*. 2020; **75**: 104952.

21. Tang J, He C, Tang J *et al.* A reliable all-2D materials artificial synapse for high energy-efficient neuromorphic computing. *Adv Funct Mater*. 2021; **31**: 2011083.

22. Luo Z, Xie Y, Li Z *et al.* Plasmonically engineered light-matter interactions in Au-nanoparticle/MoS_2 heterostructures for artificial optoelectronic synapse. *Nano Res*. 2021; **15**: 3539-3547.

23. Xie Z, Zhuge C, Zhao Y *et al.* All-solid-state vertical three-terminal N-type organic synaptic devices for neuromorphic computing. *Adv Funct Mater*. 2022; **32**: 2107314.

24. Yu T, Zhao Z, Jiang H *et al.* $MoTe_2$-based low energy consumption artificial synapse for neuromorphic behavior and decimal arithmetic. *Mater Today Chem*. 2023; **27**: 101268.

25. Assi DS, Huang H, Karthikeyan V *et al.* Quantum topological neuristors for advanced neuromorphic intelligent systems. *Adv Sci*. 2023; **10**: 2300791.

26. Zhu M, Yu Z, Hu G *et al.* A TaO_x/TiO_y bilayer memristor with enhanced synaptic features for neuromorphic computing. *Adv Electron Mater*. 2024; **10**: 2400008.
